# Robust and interpretable AI-guided marker for early dementia prediction in real-world clinical settings

**DOI:** 10.1016/j.eclinm.2024.102725

**Published:** 2024-07-12

**Authors:** Liz Yuanxi Lee, Delshad Vaghari, Michael C. Burkhart, Peter Tino, Marcella Montagnese, Zhuoyu Li, Katharina Zühlsdorff, Joseph Giorgio, Guy Williams, Eddie Chong, Christopher Chen, Benjamin R. Underwood, Timothy Rittman, Zoe Kourtzi

**Affiliations:** a Department of Psychology, University of Cambridge, Cambridge, CB2 3EB, United Kingdom; b School of Computer Science, University of Birmingham, Birmingham, B15 2TT, United Kingdom; c Department of Clinical Neurosciences, University of Cambridge, Cambridge, CB2 0QQ, United Kingdom; d Helen Wills Neuroscience Institute, University of California Berkeley, Berkeley, CA, USA; e School of Psychological Sciences, College of Engineering, Science and the Environment, University of Newcastle, Newcastle, New South Wales, Australia; f Wolfson Brain Imaging Centre, Department of Clinical Neurosciences, University of Cambridge, Cambridge, CB2 0QQ, United Kingdom; g Memory, Aging, and Cognition Center, Department of Pharmacology, Yong Loo Lin School of Medicine, National University of Singapore, Singapore; h Department of Psychiatry, University of Cambridge, Cambridge, CB2 0SZ, United Kingdom; i Cambridgeshire and Peterborough NHS Foundation Trust, Windsor Unit, Fulbourn Hospital, Cambridge, CB21 5EF, United Kingdom

**Keywords:** Dementia prediction, Prognosis, Machine learning, Cognition, Brain imaging

## Abstract

**Background:**

Predicting dementia early has major implications for clinical management and patient outcomes. Yet, we still lack sensitive tools for stratifying patients early, resulting in patients being undiagnosed or wrongly diagnosed. Despite rapid expansion in machine learning models for dementia prediction, limited model interpretability and generalizability impede translation to the clinic.

**Methods:**

We build a robust and interpretable predictive prognostic model (PPM) and validate its clinical utility using real-world, routinely-collected, non-invasive, and low-cost (cognitive tests, structural MRI) patient data. To enhance scalability and generalizability to the clinic, we: 1) train the PPM with clinically-relevant predictors (cognitive tests, grey matter atrophy) that are common across research and clinical cohorts, 2) test PPM predictions with independent multicenter real-world data from memory clinics across countries (UK, Singapore).

**Findings:**

PPM robustly predicts (accuracy: 81.66%, AUC: 0.84, sensitivity: 82.38%, specificity: 80.94%) whether patients at early disease stages (MCI) will remain stable or progress to Alzheimer's Disease (AD). PPM generalizes from research to real-world patient data across memory clinics and its predictions are validated against longitudinal clinical outcomes. PPM allows us to derive an individualized AI-guided multimodal marker (i.e. predictive prognostic index) that predicts progression to AD more precisely than standard clinical markers (grey matter atrophy, cognitive scores; PPM-derived marker: hazard ratio = 3.42, p = 0.01) or clinical diagnosis (PPM-derived marker: hazard ratio = 2.84, p < 0.01), reducing misdiagnosis.

**Interpretation:**

Our results provide evidence for a robust and explainable clinical AI-guided marker for early dementia prediction that is validated against longitudinal, multicenter patient data across countries, and has strong potential for adoption in clinical practice.

**Funding:**

10.13039/100010269Wellcome Trust, 10.13039/501100000288Royal Society, 10.13039/501100002283Alzheimer’s Research UK, Alzheimer’s Drug Discovery Foundation Diagnostics Accelerator, 10.13039/100012338Alan Turing Institute.

**Research in context**

**Evidence before this study**

We searched PubMed, bioRxiv, MedRxiv, ArXiv, IEEE Xplore, ScienceDirect (from the inception of the databases to date) for machine learning (ML) models of early dementia prediction from routinely-collected clinical data, focusing on models that predict future cognitive decline and are validated against longitudinal data. Most ML models: 1) have limited generalizability to clinical settings as they are developed on research data that are invasive, costly and limited in representing demographics and comorbidities in clinical populations, 2) stratify individuals based on cross-sectional (i.e. at different disease stages) rather than longitudinal data, using clinical labels that are poorly constrained and may result in misclassification.

**Added value of this study**

We bridge the gap between AI and clinical translation, by building a robust and interpretable predictive prognostic model (PPM) that: 1) introduces a transparent trajectory modelling approach to reliably predict future cognitive health from routinely-collected, low-cost multimodal data, 2) generalizes from research cohort to multicenter real-world patient data from memory clinics (UK, Singapore). We demonstrate that the PPM robustly predicts whether patients at early disease stages (MCI) will remain stable or progress to AD, providing an individualized prognostic index of future cognitive decline. This PPM-derived multimodal marker: 1) reliably predicts future cognitive decline as validated against longitudinal clinical outcomes, 2) is a more precise predictor of conversion to AD than standard clinical markers (i.e. grey matter atrophy, cognitive data) alone or clinical diagnosis, enhancing its potential for translation to real-world clinical settings.

**Implications of all the available evidence**

Translating our clinical AI-guided tool for early dementia prediction in real-world clinical settings has potential to: 1) reduce misdiagnosis at early stages of dementia, improving patient wellbeing, 2) standardize diagnosis across memory clinics, reducing inequalities in healthcare, 3) reduce the need for invasive and costly diagnostic tests, 4) allow scarce resources to be targeted to those who need them the most, 5) improve treatment outcomes when interventions (lifestyle changes or new pharmacological targets) may have a chance to work best.

## Introduction

Dementia poses a significant global healthcare challenge, impacting over 55 million people worldwide at an estimated annual cost of $820 billion and a threefold rise expected in 50 years.^1^ Alzheimer's disease (AD) stands as the predominant cause of dementia, accounting for 60–80% of cases.^2^ Predicting who will develop AD early has major implications for clinical management and treatment. Recent positive phase three clinical trial results (i.e. lecanemab, donanemab)^3^^,^^4^ highlight the critical need for early detection when treatments may be maximally effective.^5^^,^^6^ Yet, we still lack effective tools for early dementia diagnosis and prognosis. Standard memory tests lack sensitivity, especially at early disease stages, and most patients do not have access to more specific positron emission tomography (PET) scans or lumbar punctures (i.e. cerebrospinal fluid biomarkers). These invasive or costly biomarkers are therefore not included in routine clinical practice, leading to significant inequalities in healthcare. As a result, up to a third of patients may be misdiagnosed and others diagnosed too late for treatment to be effective (e.g.^7^).

Maturing analytical techniques provide a turning point in addressing these challenges and improving early prediction and prognosis of dementia using lower-cost, less invasive assessments. Despite, the increasing success of Artificial Intelligence (AI) models based on machine learning (ML) algorithms in stratifying individuals, translating models to clinical practice is hampered due to the following main reasons.^8^, ^9^, ^10^, ^11^ First, ML models developed using research cohort data alone^8^ may be limited in representing demographics and medical comorbidities in clinical populations. Second, research cohort data are rich (e.g. biomarkers) and structured; in contrast, data in real-world settings are collected using low-cost and less sensitive measures and may be missing and “messy”, due to lack of standardized methods for assessment across healthcare providers. As a result, ML models trained on research cohort data may fail to generalize when tested on real-world patient data. Third, many of the existing ML models focus on stratifying individuals in a cross-sectional manner (i.e. at different disease stages); yet, predicting individualized health trajectories is key for the prognosis of neurodegenerative disorders that span a continuum from health to disease.^12^

To address these limitations that hamper clinical translation, we build a robust and interpretable predictive prognostic model (PPM) that extends beyond binary patient classification approaches and predicts whether and how fast individuals at early stages of the disease (Mild Cognitive Impairment, MCI) or pre-symptomatic (Cognitive Normal, CN) may progress to AD.^13^^,^^14^ We demonstrate the clinical utility of our approach and potential for adoption in clinical practice by 1) training the PPM on multimodal baseline (first assessment) non-invasive and low-cost data that are typically used in the clinical assessment of dementia (cognitive tests, structural MRI) to enhance scalability, 2) testing the PPM on independent (out-of-sample) real-world patient data from memory clinics to assess generalizability, 3) validating PPM prognosis against longitudinal diagnosis (i.e. conversion to AD) in real-world patient data. We demonstrate that the PPM robustly predicts whether patients at early disease stages (MCI) will remain stable or progress to AD, as validated against longitudinal clinical outcomes. Importantly, the PPM— trained on multimodal research cohort data (USA) — generalizes to real-world patient data from memory clinics in distinct settings (UK, Singapore). We next derive an AI-guided multimodal marker of future cognitive health; that is, an individualized PPM-derived prognostic index of cognitive decline over time. We demonstrate that this clinical AI-guided marker predicts conversion to AD more precisely than standard clinical markers (i.e. grey matter atrophy, cognitive data) or clinical diagnosis at initial assessment, demonstrating its potential to reduce misdiagnosis and help clinicians standardize diagnosis and assign patients to clinical management pathways that best meet their needs.

## Methods

### PPM training and test samples

**Fig. 1:**
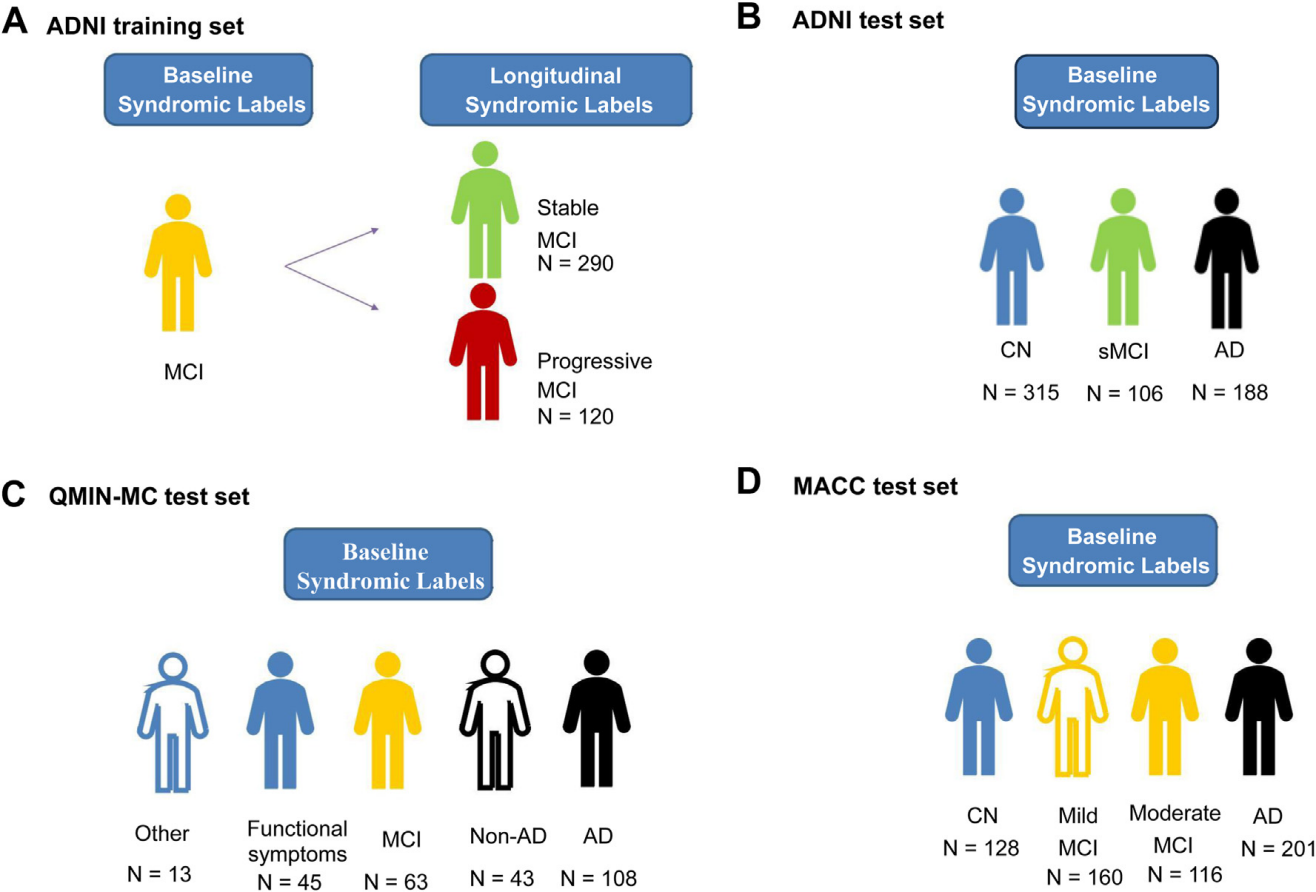
**Overview of Cohort Data used for PPM training and validation: A.** research cohort data: Alzheimer's Disease Neuroimaging Initiative (ADNI) data used as training Set, **B.** independent ADNI data used for validation, **C.** memory clinic data used as test data: Quantitative MRI of Brain Structure and Function in NHS Memory Clinics (QMIN-MC), **D.** memory clinic data used as test data: Memory Ageing & Cognition Centre at the National University of Singapore (MACC).

We used data (Fig. 1; Supplementary Information: PPM training and test samples) from: 1) a research cohort (the Alzheimer's Disease Neuroimaging Initiative, ADNI) for PPM training with within-sample cross-validation (n = 410) and out-of-sample validation (n = 609), 2) two clinical cohorts as independent test datasets for out-of-sample validation: Quantitative MRI of Brain Structure and Function in NHS Memory Clinics (QMIN-MC, n = 272; Figure S1, Table S1); Memory Ageing & Cognition Centre at the National University of Singapore dataset (MACC, n = 605).

These datasets differ in patient demographics and data collection tools (e.g. 1.5 T and 3T MRI scanners for ADNI, 3T scanners for QMIN-MC, MACC) allowing us to test PPM interoperability across memory clinics and countries. In particular, for ADNI individuals were selected based on specific criteria related to amnestic MCI and Alzheimer's disease and MRI data were collected across MRI acquisition sites in the US. In contrast, for the QMIN-MC and MACC data were collected from representing neurology-led and psychiatry-led memory services in the UK and Singapore, respectively. Thus, these patient cohorts are likely to reflect higher real-world diversity in patient demographics and comorbidities typically encountered in clinical practice, compared to research cohorts with more selective recruitment criteria (e.g. ADNI).

### Predictive prognostic modelling

We have developed a trajectory modelling approach based on Generalized Metric Learning Vector Quantization (GMLVQ)^15^^,^^16^ that leverages multimodal data to make predictions about future cognitive decline at early dementia stages by iteratively adjusting class-specific prototypes and learning class boundaries (Supplementary Information: Predictive prognostic model). GMLVQ incorporates a full metric tensor to provide a robust distance measure (metric) tuned to the classification task. The metric tensor can naturally handle specific feature scaling and pairwise task-conditional dependencies of the input features. Dominant diagonal elements of the metric tensor identify key univariate predictors, while the off-diagonal terms reveal pairwise feature interactions contributing to this classification task.

We trained GMLVQ models to discriminate stable MCI (sMCI: individuals who consistently received an MCI diagnosis within a 3-year period) vs. progressive MCI (pMCI: individuals who progressed to AD within a 3-year period)^13^ using baseline ADNI data (medial temporal lobe grey matter (GM) density^13^^,^^14^ (see Supplementary Information: MRI analysis: extracting medial temporal grey matter density), The Addenbrooke's Cognitive Examination Revised memory scale (ACE-R memory), Mini-Mental State Examination (MMSE)) that corresponded to available datatypes in clinical patient cohorts (QMIN-MC, MACC) that served as test datasets. All data were adjusted for potential confounding covariates (i.e. age, sex, and education). Following previous work,^13^^,^^14^ we performed hyper-parameter tuning for the model using a nested cross-validation approach,^17^ considering two hyper-parameters. To evaluate the model's performance, we employed 10 iterations of a 10-fold cross-validation.^17^ To mitigate any potential biases due to class imbalance in the dataset (sMCI, n = 290; pMCI, n = 120), we resampled the data to generate balanced classes. For each training-fold, we repeatedly (n = 400) randomly down-sampled the majority class (i.e. sMCI) to match the size of the minority class (i.e. pMCI). Further, we introduced ensemble learning,^18^ combining multiple models (n = 400) for robust learning of unbalanced classes that is typical in real-world clinical data (i.e. patient groups are likely to vary in size). We selected the top 20% (n = 80) models based on their training set performance and estimated the class balanced accuracy based on a) majority vote, i.e. the class label that receives the most votes from the ensemble models is selected as the final prediction,^18^ b) the average performance across the selected classifiers.^19^ This ensemble learning approach with cross-validation helps mitigate potential individual model biases, resulting in more robust and accurate predictions.^20^^,^^21^

### Statistical analysis

We tested for data normality using the Shapiro–Wilk test. As the data (i.e. PPM-derived prognostic index) were not normally distributed (ADNI, PPM trained on MRI and cognitive data, W (609) = 0.881, p < 0.001; ADNI, PPM trained on cognitive data, W (609) = 0.862, p < 0.001; QMIN-MC, PPM trained on MRI and cognitive data, W (272) = 0.980, p < 0.001; QMIN-MC, PPM trained on cognitive data, W (272) = 0.967, p < 0.001; MACC, PPM trained on MRI and cognitive data, W (605) = 0.959, p < 0.001; MACC, PPM trained on cognitive data, W (605) = 0.954, p < 0.001), we used Kruskal–Wallis H test Bonferroni corrected (p < 0.05) to examine differences in the PPM-derived prognostic index across the different patient groups. We used Spearman's rank correlation to test whether the relationship between the PPM-derived prognostic index and the rate of future cognitive decline (CDR change) was significant. We used Steiger Z to compare correlations and the DeLong test to compare AUCs (area under ROC) across models.

Further, to test whether the PPM-derived prognostic stratification (i.e. stable, slowly progressive, rapidly progressive) from baseline (i.e. first assessment) patient data is validated against longitudinal data (i.e. clinical diagnosis indicating conversion to AD), we conducted a survival analysis for the MACC sample over 6 years (note, there are no longitudinal assessments yet available for QMIN-MC). We used logrank test to compare the relative risk of ‘conversion to AD’ for patient groups given different PPM-derived prognostic stratification. We used a multivariate Cox proportional hazards regression analysis to identify independent predictors of ‘conversion to AD’ with hazard ratios at 95% confidence intervals (CIs). For more details on statistical analysis methods, see Supplementary Information (Statistical analyses).

### Role of the funding source

The funders of the study had no role in study design, data collection, data analysis, data interpretation, or writing of the manuscript. LYL, DV, MCB, MM, ZL, KZ, JG, EC, CC, BRU, TR and ZK had access to the dataset. All authors accept responsibility to submit for publication.

## Results

### PPM-guided diagnosis: model training and within sample validation

We trained the PPM on baseline data (i.e. first assessment) from ADNI (n = 410; Fig. 1) to discriminate sMCI (n = 290) from pMCI (n = 120) patients, using ACE-R memory, MMSE, and GM density. These data types were shown to have the highest contribution compared to a range of features in discriminating sMCI vs. pMCI (Figure S2, Table S2).

**Fig. 2:**
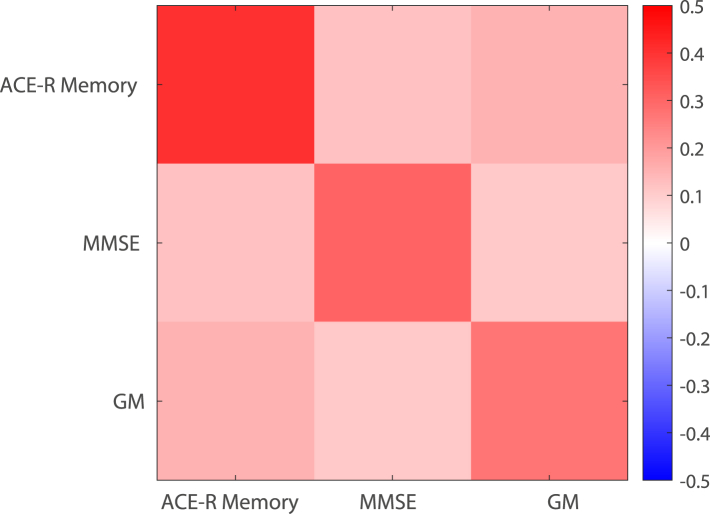
**PPM Metric Tensor for sMCI vs. pMCI classification:** PPM metric tensor generated using ACE-R memory, MMSE, and temporal lobe GM density. The color scale represents predictive values for each cell in the metric tensor, with diagonal terms summing to 1. The diagonal terms show strong contribution of ACE-R memory (0.41 [0.37, 0.44]) compared to MMSE (0.31 [0.29, 0.33]) and GM density (0.29 [0.26, 0.32]). Positive off-diagonal terms indicate interactions between ACE-R memory, MMSE, and GM density.

Our results showed 81.66% [81.23, 82.09] classification accuracy (AUC: Area Under Curve: 0.84; [0.83, 0.84]) with sensitivity of 82.38% [81.69, 83.07] and specificity of 80.94% [80.57, 81.29] (Table S2). Interrogating the PPM metric tensors (Fig. 2) shows that ACE-R memory was the most discriminative feature (i.e. feature with highest weight = 0.40 [0.37, 0.44]), compared to MMSE (weight = 0.31 [0.29, 0.33]) and GM density (weight = 0.29 [0.26, 0.32]). Positive off-diagonal terms indicate a positive interaction between ACE-R memory, MMSE and GM density, providing evidence for the role of interactive multimodal features in accurately distinguishing between sMCI and pMCI.

We next asked whether structural MRI is necessary for MCI patient stratification. Our results showed that when PPM was trained with ACE-R memory and MMSE alone, performance was similar to the model trained with both cognitive and MRI data (accuracy = 80.03% [79.71, 80.35], AUC = 0.83 [0.82, 0.83], sensitivity = 80.58% [79.79, 80.35], specificity = 79.48% [78.86, 80.10]; Table S2). Comparing between models did not show a significant difference in AUC (DeLong test: p = 0.516). However, performance was lower when the PPM was trained with individual features alone (Table S2; DeLong test: MMSE (p < 0.001), GM density (p < 0.001); ACE-R Memory (marginal, p = 0.073)), suggesting that PPM achieves best stratification when leveraging the higher-order multivariate interactions across multimodal data. If structural MRI data is not available, PPM maintains good classification performance when data from multiple complementary cognitive tests that capture memory performance are available.

### PPM for prognosis

To extend the PPM beyond binary classification (sMCI vs. pMCI) into a trajectory modelling approach that predicts future cognitive decline, we used a scalar projection method^13^ to generate a PPM-derived prognostic index (Supplementary Information: GMLVQ—Scalar Projection). Leveraging an ensemble of GMLVQ classifiers, we averaged the metric tensors and class prototypes from the top 20% of models (80 classifiers) resulting in similar performance (accuracy 81.40% [80.22, 82.57]) as majority voting (Table S2). We next extracted the scalar projection for each individual in an independent ADNI sample (out-of-sample validation, n = 609; cognitive normal individuals, n = 315, patients with MCI, n = 106, patients with AD, n = 188). This PPM-derived prognostic index indicates the distance of an individual from the stable MCI prototype (i.e. higher index indicates higher risk of future cognitive decline), allowing individualized prognosis beyond binary clinical labels.

**Fig. 3:**
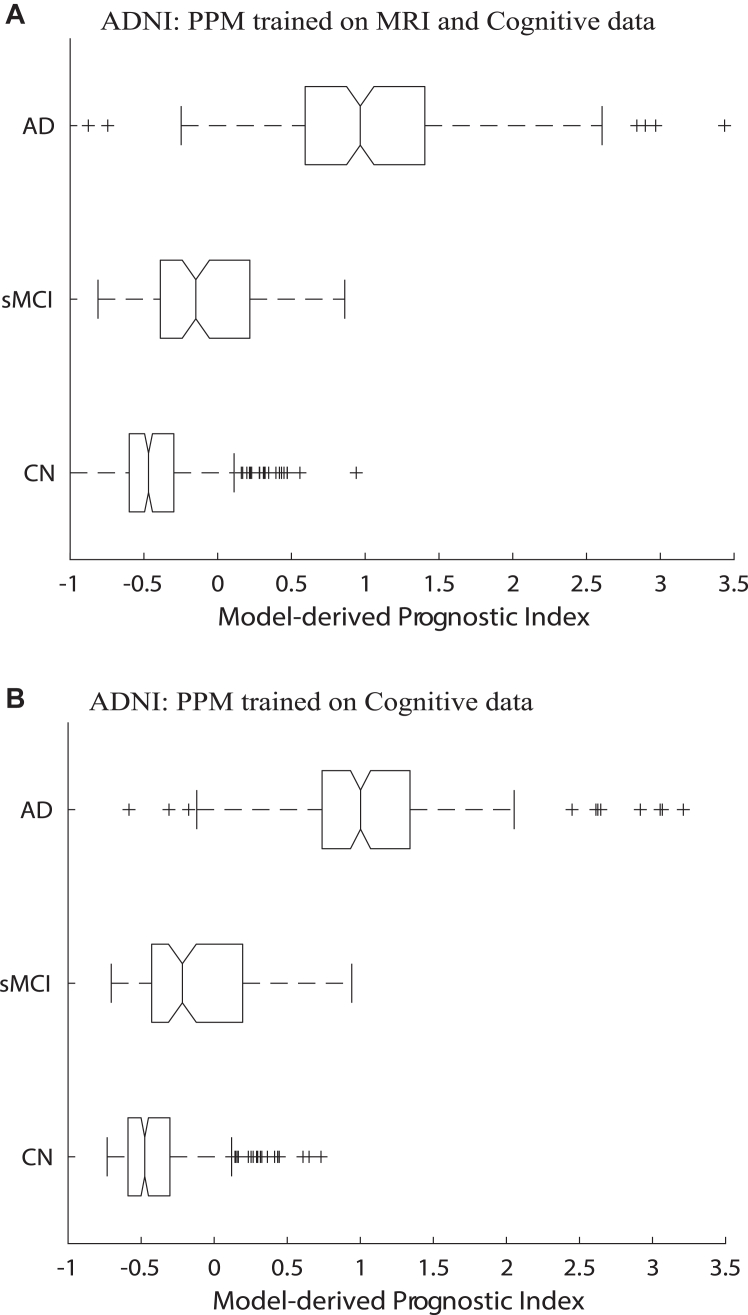
**PPM-derived prognostic index for ADNI Validation Set:** Box plots of PPM-derived prognostic index for individuals (Cognitive Normal, MCI, AD) from the ADNI validation set derived from PPM trained with: **A.** ACE-R memory, MMSE and GM density, **B.** ACE-R memory and MMSE. The solid black line in the box plots indicates the median, solid black box represents the 25th to 75th percentile, the black horizontal lines represent the range of the data, crosses are outliers from the distribution, and non-overlapping notches indicate significantly different medians (p < 0.05). PPM-derived prognostic index below 0 indicates stable, above 1 indicates rapidly progressive, and between 0 and 1 indicates slowly progressive individuals.

Our results demonstrate that the PPM-derived prognostic index is clinically relevant for predicting cognitive health trajectories. First, we show that the prognostic index derived from PPM trained on cognitive and MRI data was significantly different across groups (Fig. 3; Kruskal–Wallis H test χ(2) = 351.68, p < 0.001) with significantly higher index (Bonferroni corrected) for AD vs. MCI and CN (p < 0.001), MCI vs. CN (p < 0.001). Regressing out education from GM density and cognitive data to control for differences across individuals due to education, showed similar results; that is, the PPM-derived prognostic index was significantly different across groups (Kruskal–Wallis H test, χ(2) = 372.62, p < 0.001) with significantly higher index (Bonferroni corrected) for AD vs. MCI and CN (p < 0.001), MCI vs. CN (p < 0.001). Further, we observed similar results when the prognostic index was derived from the PPM trained on cognitive data alone (χ(2) = 366.37, p < 0.001; post-hoc caparisons (Bonferroni corrected) for AD vs. MCI and CN (p < 0.001), but not for MCI vs. CN (p = 0.221)).

Second, we show that the PPM-derived prognostic index relates to future cognitive decline (Figure S3). Spearman's rank correlation showed that the prognostic index derived from PPM trained on cognitive and MRI data relates significantly to the rate of CDR change (after regressing out age) across groups (rho = 0.54, p < 0.001, 95% CI [0.48, 0.60]). This correlation was significant for CN (rho = 0.31, p < 0.001 [0.21, 0.41]), MCI (rho = 0.25, p = 0.01 [0.06, 0.42]), AD (rho = 0.27, p < 0.001 [0.11, 0.41]). We observed similar results when the PPM was trained on cognitive data alone; that is, significant correlation across groups (rho = 0.52, p < 0.001 [0.46, 0.58]), for CN (rho = 0.25, p < 0.001 [0.15, 0.36]), MCI (rho = 0.23, p = 0.02 [0.04, 0.40]) and AD (rho = 0.21, p = 0.01 [0.05, 0.36]). Comparing these correlations for each group between models (PPM trained on MRI and cognitive data vs. cognitive data alone) showed significantly stronger effects for the former than the latter (Stegler's Z; CN: z = 1.70, p = 0.04; but not for MCI: z = 0.37 (n = 106), p = 0.36 and AD, z = 1.152 (n = 149), p = 0.13), suggesting that adding GM density enhances PPM sensitivity in predicting future cognitive decline at early or pre-symptomatic stages.

### Translating PPM from research to clinical data: out of sample validation

To test the interoperability and clinical utility of the PPM, we tested the model trained on research cohort data from ADNI with two independent data sets from real-world patient cohorts (Fig. 1; QMIN-MC, n = 272; MACC, n = 605).

**Fig. 4:**
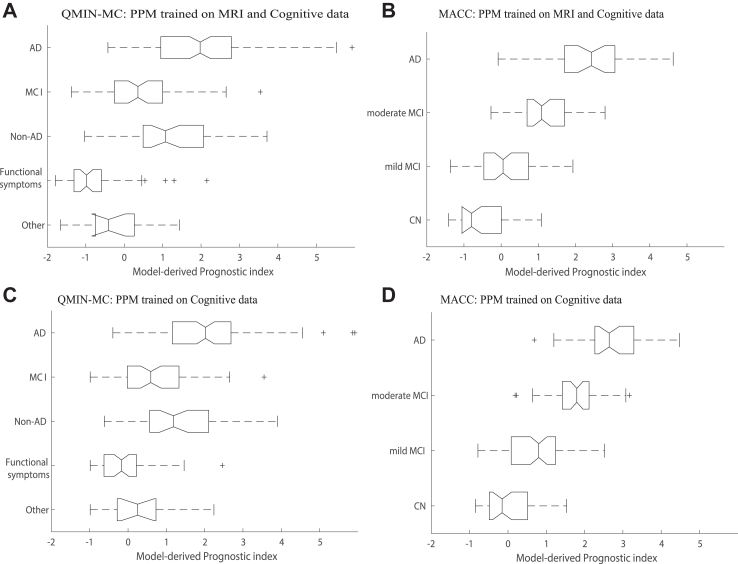
**PPM-derived prognostic index for clinical test data:** Box plots of PPM-derived prognostic index for individuals from QMIN-MC validation set (**A, C**; AD, MCI, Non-AD, Functional symptoms, Other) and the MACC (**B, D**; AD, Moderate MCI, mild MCI, cognitive normal) derived from PPM trained with ACE-R memory, MMSE and GM density (**A, B**) or ACE-R memory and MMSE (**C, D**). The solid black line in the box plots indicates the median, solid black box represents the 25th to 75th percentile, the black horizontal lines represent the range of the data, crosses are outliers from the distribution, and non-overlapping notches indicate significantly different medians (p < 0.05). PPM-derived prognostic index below 0 indicates stable, above 1 indicates rapidly progressive, and between 0 and 1 indicates slowly progressive individuals.

For both QMIN-MC and MACC, we extracted the PPM-derived prognostic index for each individual using baseline data (i.e. data from the first clinical assessment and MRI scan). We then tested whether the predicted PPM-derived index of cognitive decline relates to clinical diagnosis. For both QMIN-MC (Fig. 4A, χ(4) = 127.10, p < 0.001) and MACC (Fig. 4B, χ(3) = 435.74, p < 0.001) patients, the PPM-derived prognostic index was significantly different across groups. In particular, when the PPM was trained with cognitive and MRI data, the PPM-derived index was significantly higher (Bonferroni post-hoc comparisons) for AD vs. other groups (p < 0.001), MCI vs. functional/attentional memory symptoms (p < 0.001), moderate MCI vs. mild MCI or CN (p < 0.001), and mild MCI vs. CN (p < 0.001). Regressing out education from GM density and cognitive data to control for differences across individuals due to education, showed similar results; that is, the PPM-derived prognostic index was significantly different across groups (Kruskal–Wallis H test, QMIN-MC: (χ(4) = 62.73, p < 0.001); MACC (χ(3) = 305.37, p < 0.001)) with significantly higher index (Bonferroni corrected) for AD vs. other groups (MCI, functional/attentional memory symptoms, others, p < 0.001; non-AD neurodegenerative diseases, p = 0.022), moderate MCI vs. mild MCI or CN (p < 0.001), and mild MCI vs. CN (p = 0.024). Further, we observed similar results when the PPM was trained on cognitive data alone (Fig. 4C; QMIN-MC, χ(4) = 106.45, p < 0.001; MACC, χ(3) = 437.90, p < 0.001). Taken together, these results provide evidence for the clinical utility of our PPM trajectory modelling approach. In particular, the PPM generalizes to real-world clinical data from two independent samples, stratifies patients based on non-invasive (MRI, cognitive data) data based on first assessment and makes predictions for future cognitive health that relate to clinical diagnosis.

### Stratifying individuals based on the PPM-derived prognostic index

To enhance the interpretability and clinical utility of the PPM, we developed a methodology for stratifying individuals based on the PPM-derived prognostic index. We used multinomial logistic regression to capture the relationship of the PPM-derived prognostic index to the rate of cognitive decline (i.e. future MMSE change) and determine boundaries for quartile classes that differ in likelihood of disease progression. We scaled the boundaries so that PPM-derived prognostic index indicates individuals who are more likely to: 1) remain stable (PPM index below 0), 2) experience rapid progression (PPM index higher than 1), 3) experience slower progression (PPM index between 0 and 1) (Fig. 3, Fig. 4).

Our results demonstrate that this PPM-derived stratification of patients is clinically relevant. In particular, for ADNI test data (Fig. 3), most cognitive normal individuals (90.5%) were classified as stable, 58.5% of individuals with MCI diagnosis were stratified as stable and 42.5% as slowly progressive, while most AD individuals were stratified as progressive (96.2%). For QMIN-MC (Fig. 4A), most individuals diagnosed with AD (97.2%; n = 108) and non-AD neurodegenerative disorders (90.7%; n = 43) were stratified as progressive, while most individuals diagnosed with MCI (n = 63) were stratified as stable (34.9%) or slowly (39.7%) progressive. For MACC (Fig. 4B), most individuals diagnosed with AD (n = 201) or moderate MCI (n = 116) were stratified as rapidly progressive (92.5%, 59.5%, respectively). In contrast, most individuals diagnosed with mild MCI (n = 160) were stratified as slowly progressive (35.0%) or stable (50.0%) and most individuals diagnosed as cognitive normal (CN, n = 128) were stratified as stable (88.3%).

It is interesting to note that the PPM stratified some individuals diagnosed with MCI as stable (QMIN-MC: 34.9%; MACC: 31.2%), consistent with previous studies (e.g.^7^) reporting up to 35% misdiagnosis at early dementia stages, suggesting that our modelling approach has potential to reduce misdiagnosis. Further, including biological data may facilitate reducing false positives; that is, more patients were stratified as stable when the PPM was trained on both cognitive and MRI data (QMIN-MC, functional symptoms or other cognitive disorders: 79.3%; MACC, CN and mild MCI: 67.0%) than cognitive data alone (QMIN-MC: 60.3%; MACC: 41.7%).

Finally, we conducted an ordinal regression to test the rate at which the PPM index discriminates between groups (AD; MCI; MACC: CN; QMIN-MC: Functional Symptoms, Others) across the QMIN-MC and MACC validation samples. Our results showed that when the PPM was trained on MRI and cognitive data, the PPM-derived prognostic index discriminated between groups at an overall rate of 0.70 (i.e. 0.75 for AD; 0.68 for MCI; 0.66 for CN compared to other groups). When the PPM was trained on cognitive data alone, the overall rate was 0.69 (i.e. 0.75 for AD; 0.68 for MCI; 0.63 for CN, compared to other groups).

### Comparing PPM-derived prediction to standard clinical assessments and statistical approaches

To assess the clinical validity and utility of the PPM, we conducted additional analyses comparing the PPM against predictions derived from: 1) assessments typically available in clinical practice, 2) simpler ML models (logistic regression), 2) more conventional statistical methods (multiple regression). Our results show that PPM has higher accuracy and sensitivity than these modelling approaches.

First, in most clinical settings, MCI vs. AD diagnosis is based on age, education and cognitive scales (e.g. MMSE). Training and testing the PPM with these datatypes results in 74.64% accuracy, sensitivity: 75.00%, specificity: 75.04%. In contrast, training and testing the PPM with cognitive data (ACE-R memory, MMSE) and GM density achieved significantly (DeLong test: p < 0.001) higher accuracy (81.36%, sensitivity: 81.92%, specificity: 80.76%).

Second, PPM trained on ADNI ACE-R mem, MMSE and GM density performs (class balanced accuracy for 10 repetitions of 10 fold cross-validation) better than a logistic regression model when tested on the ADNI validation sample. In particular, PPM showed accuracy: 81.66 ± 0.25%, AUC: 0.84, sensitivity: 82.38%, specificity: 80.94%. In contrast, a logistic regression model with class balanced accuracy (10 repetitions of 10 fold cross-validation) showed accuracy: 73.41 ± 0.95%, AUC: 0.82 with sensitivity: 62.98%, specificity: 86.42%. That is, PPM showed both higher accuracy and sensitivity than a simple logistic regression model (t-test, t (198) = 8.86, p < 0.001; permutation testing showed that PPM outperforms logistic regression 92% of the times).

Third, we trained a multiple regression model on ADNI training data using ACE-R mem, MMSE and GM density to predict future cognitive decline (rate of MMSE change). Testing this multiple regression model on the ADNI validation dataset showed Root Mean Squared Error (RMSE) of 1.81. In contrast, using the PPM-derived prognostic index to predict future cognitive decline in the ADNI validation sample showed significantly (Diebold–Mariano Test: T = −2.043, p = 0.04) lower RMSE (1.74). Further, using the same multiple regression model to predict future cognitive decline (rate of MMSE change) in MACC based on the PPM-derived prognostic index showed significantly (Diebold–Mariano Test: T = −9.136, p < 0.001) lower RMSE (1.60) than using ACE-R mem, MMSE and GM density as predictors (2.17). These results, suggest our PPM-derived marker predicts future cognitive decline more precisely than standard clinical assessments.

### Longitudinal validation of PPM-derived prognosis

We tested whether the PPM-derived stratification based on the prognostic index (i.e. stable, slowly progressive, and rapidly progressive) from baseline (i.e. first assessment) patient data is validated against future clinical outcomes (i.e. longitudinal data on clinical diagnosis indicating conversion to AD). We used longitudinal data available for MACC over 6 years. We conducted survival analyses to investigate the risk of converting to AD for CN and MCI patients with longitudinal diagnoses (n = 387) that were stratified using the PPM-derived prognostic index at baseline as stable (n = 189), slowly (n = 111) or rapidly (n = 87) progressive.

**Fig. 5:**
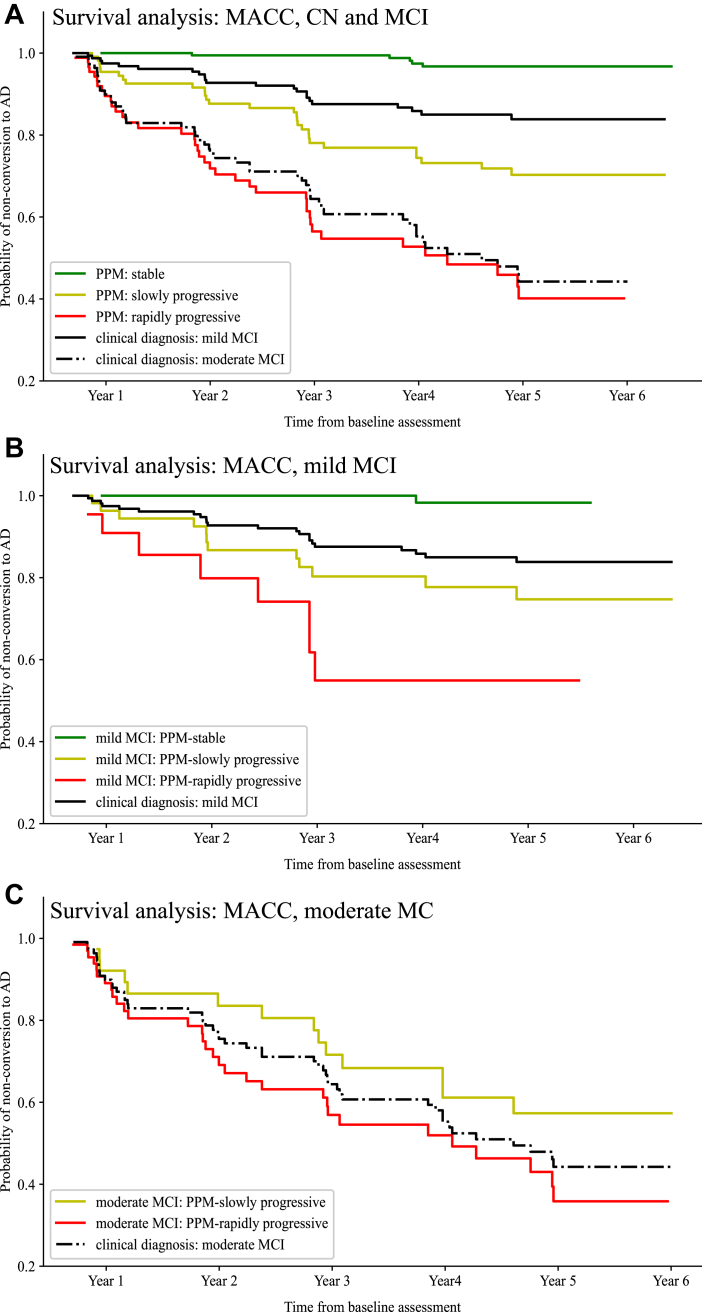
**Survival analysis for MACC data:** Survival curves (Kaplan–Meier estimator) are fit to longitudinal clinical diagnosis data for MACC CN and MCI individuals **A.** Survival curves for all MACC patients with longitudinal clinical diagnoses (n = 387) stratified based on clinical diagnosis at first assessment (mild MCI: solid black line; moderate MCI: dashed black line) or the PPM-derived index (stable: green, n = 189; slowly progressive: yellow, n = 111; rapidly progressive: red, n = 87). **B.** Survival curves for patients with clinical diagnoses of mild MCI (n = 153) stratified based on clinical diagnosis (mild MCI: solid black line) at first assessment or the PPM-derived index (stable: green, n = 75; slowly progressive: yellow, n = 56; rapidly progressive: red, n = 22). **C.** Survival curves for patients with clinical diagnoses of moderate MCI (n = 108) stratified based on clinical diagnosis (moderate MCI: dashed black line) at first assessment or the PPM-derived index (slowly progressive: yellow, n = 38; rapidly progressive: red, n = 65). We did not fit a survival curve for moderate MCI patients stratified by the PPM as stable due to small sample size (n = 5).

First, we validate PPM-derived predictions at first assessment against longitudinal clinical diagnosis. Fig. 5A shows that individuals who were predicted by the PPM to remain stable rarely (0.5%) converted to AD, while 18.9% of individuals predicted to progress slowly vs. 41.4% of individuals predicted to progress rapidly converted to AD within a 3-year period (Kaplan–Meier graphical display; survival rates per year based on annual assessments). In contrast, based on clinical diagnosis, 3.2% of CN, 11.8% of mild MCI, and 30.6% of moderate MCI individuals converted to AD within 3 years. Kaplan–Meier survival analysis showed an overall ‘non-conversion to AD’ advantage for the stable group (logrank test, stable group vs. slowly progressive group, χ^2^ = 41.31 on df = 1, Bonferroni-adjusted p < 0.01), while a higher risk of conversion to AD for rapidly progressive (logrank test, slowly progressive vs. rapidly progressive, χ^2^ = 13.64 on df = 1, Bonferroni-adjusted p < 0.01).

Second, we demonstrate that the PPM-derived prognostic index is a stronger predictor of conversion to AD risk than standard clinical markers (i.e. grey matter atrophy, cognitive decline) at baseline. In particular, a multivariate Cox Proportional Hazard (CoxPH; continuous variable) model^22^ showed that the PPM-derived prognostic index (hazard ratio = 3.42 [1.36, 8.60], p = 0.015) —rather than GM density (p = 0.24) or MMSE (p = 0.92), was a significant predictor of conversion to AD.

Third, we demonstrate that stratifying patients at baseline based on the PPM is more precise than standard stratification based on clinical diagnosis. In particular, a multivariate CoxPH^22^ model (categorical variable) showed that stratification based on the PPM-derived marker (i.e. stable, slowly vs. rapidly progressive) or clinical diagnosis (i.e. CN, mild MCI, moderate MCI) were significant predictors of conversion to AD, with the PPM-derived marker showing a higher hazard ratio (hazard ratio = 2.84 [1.88, 4.29], p < 0.01) than clinical diagnosis (hazard ratio = 1.98 [1.23, 3.18], p < 0.01).

Further, using Kaplan–Meier survival analysis, we compared the risk of conversion to AD when individuals were stratified based on a) the PPM-derived prognostic index, b) clinical diagnosis at baseline, that is, mild MCI (Fig. 5B) or moderate MCI (Fig. 5C). Our results showed that stratifying individuals based on PPM compared to clinical diagnosis supports more precise prediction of conversion to AD risk. Fig. 5B shows that nearly half of the patients (49.0%) with mild MCI who were stratified by the PPM as stable did not convert to AD within a 3-year window; in contrast, stratifying based on clinical diagnosis predicts that individuals with mild MCI are at risk of converting to AD within three years of first assessment (year 1, 2.6%; year 2, 7.2%; year 3, 11.7%). Our results showed that the ‘conversion to AD’ risk for all individuals with clinical diagnosis of mild MCI (Fig. 5B) was significantly higher than individuals with mild MCI who were stratified by the PPM as stable (49.0%; logrank test, χ2 = 9.29 on df = 1, Bonferroni-adjusted p < 0.01) from year 1 onwards, while significantly lower than individuals with mild MCI who were stratified by the PPM as rapidly-progressive (14.4%; logrank test, χ2 = 10.62, on df = 1, Bonferroni-adjusted p < 0.01) from year 3 onwards (χ2 = 11.75, p < 0.01). This advantage of PPM-based stratification is more prominent at early stages of disease (i.e. mild MCI). That is, we didn't observe any significant differences between the overall ‘conversion to AD’ risk for all individuals with clinical diagnosis of moderate MCI (Fig. 5C) vs. individuals with moderate MCI who were stratified by the PPM as: a) slowly progressive (35.2%, logrank test, p = 0.26), b) rapidly progressive (60.2%, logrank test, p = 0.38).

Taken together, our results provide evidence for an AI-guided multimodal marker (i.e. PPM-derived prognostic index) that predicts risk of conversion-to-AD at early stages (i.e. mild MC) more precisely than standard clinical markers (i.e. grey matter atrophy, cognitive decline) or clinical diagnosis at first assessment.

## Discussion

To bridge the gap between AI and clinical translation, we built a robust and interpretable clinical-AI tool based on a predictive prognostic model (PPM) that: 1) introduces a transparent trajectory modelling approach to reliably predict future cognitive health from multimodal routinely-collected data, 2) generalizes from research cohort to real-world patient data, enhancing clinical utility and potential for adoption into healthcare, 3) delivers an AI-guided multimodal marker that supports more precise prediction of conversion to AD at early stages than standard clinical markers (i.e. grey matter atrophy, cognitive decline) or clinical diagnosis. This integrative modelling approach provides the following main advances for translating robust and responsible AI models for early dementia prediction to real-world clinical settings.

First, adopting a GMLVQ framework with ensemble learning enables us to develop a robust model^20^^,^^21^ by combining data from multiple disease-relevant modalities, rather than considering single data types. PPM harnesses the power of multimodal data to predict from routinely-collected, non-invasive and low-cost data (cognitive data, structural MRI) that may be less sensitive than biomarkers but can be collected more readily at population level. This AI-guided approach to robust early prediction at scale has strong potential to: 1) improve patient wellbeing and reduce healthcare costs as fewer patients undergo invasive and costly diagnostic tests, 2) target scarce resources to those that need them the most, 3) standardize diagnosis across memory clinics, reducing inequalities in healthcare.

Second, the GMLVQ framework allows us to develop transparent models for early dementia prediction, that is key for trusted clinical-AI solutions. In particular, interrogating the model metric tensors allows us to rank the contribution of different data types (i.e. clinically-relevant predictors) and their interactions for patient classification. This has potential to advance our understanding of disease mechanisms and enhance model interpretability, in contrast to deep learning methods that may be difficult to interpret and generalize (for review^23^).

Third, PPM extends from diagnosis to prognosis to capture individual disease trajectories. Most machine learning models for dementia prediction have focused on binary classifications^24^ based on clinical labels (e.g. CN vs AD) that are poorly constrained; as a result individual patients at the class boundary that differ only slightly in their trajectory may be misclassified.^8^ Trajectory modelling approaches that estimate time to AD conversion (e.g.^25^, ^26^, ^27^) may be limited by target uncertainty (e.g. variability in follow-up assessments, clinical diagnosis). In contrast, PPM provides a continuous index of future cognitive health from first assessment data, reducing misdiagnosis associated with clinical labels. We demonstrate that the PPM-derived prognostic index reliably predicts cognitive decline over time (i.e. changes in CDR) and is a better predictor of conversion to AD than standard clinical assessments (i.e. cognitive data, MRI scan). Thus, PPM supports precise prognostication with strong potential to impact both the diagnostic and treatment pathway, allowing patients and their families to plan for the future, facilitating clinicians to determine a personalized diagnostic and treatment pathway.

Fourth, PPM-derived prognosis (i.e. prediction of progression to AD) generalizes to independent real-world patient data from memory clinics. Lack of generalizability of ML models is a key barrier to adoption in healthcare. Validation of ML classification models against independent clinical cohorts remains limited, with a recent review^8^ reporting that most models use the same research dataset (i.e. ADNI) for training and test. Some models using patient cohort data focus on cross-sectional data and stratification across classes (e.g. AD vs. healthy controls,^28^^,^^29^). In contrast, PPM makes predictions about future progression to AD that generalize and are validated against longitudinal data in independent real-world patient cohorts. To enhance PPM generalizability and clinical utility, we take the following steps: 1) train the PPM with clinically-relevant predictors (e.g. medial temporal lobe atrophy) that are common across research and clinical cohorts, 2) implement imputation methods for harmonizing cognitive data across cohorts with missing data, 3) test the model with independent multicenter data samples from different countries (USA, UK, Singapore). Potential limitations to generalizability include the size and diversity of the population sample as well as data collection tools (cognitive tests tailored for diverse populations, MRI scanners of different magnetic field strength) used for training and testing the model. Access to larger real-world patient data across healthcare systems and countries collected using different tools will allow us to train and test the PPM on highly diverse clinical cohorts. This will ensure that PPM predictions are validated across more representative populations, enhancing global clinical utility.

Importantly, we derive a clinical-AI multimodal biomarker, validating the clinical utility of the PPM-derived index not only for diagnosis but also prognosis against longitudinal clinical outcomes. We demonstrate that the PPM-derived marker predicts conversion to AD more precisely than clinical diagnosis at early stages of disease. In particular, patients stratified as stable or slowly progressive by the PPM have lower risk of conversion to AD than when stratified based on clinical diagnosis (i.e. mild MCI) at first assessment. Thus, our clinical AI-guided marker has strong potential to help clinicians assign patients to the clinical management pathway that best meets their needs (i.e. reducing invasive diagnostic testing and hospitalization rates). For example, mild MCI patients stratified by the PPM as rapidly progressive may need to proceed with invasive diagnostic testing (e.g. PET scans) and pharmacological treatments, while patients stratified as stable and at lower risk of conversion to AD may be recommended life-style interventions and follow up at a later time. Thus, the PPM-derived marker has strong potential to aid clinicians standardize diagnosis and interventions across healthcare systems and allocate resources to those that need them the most, reducing costs and inequalities in dementia care.

Finally, scaling up the PPM to a clinical-AI tool for adoption in healthcare involves the following next steps: 1) extending to prediction of dementia subtypes (e.g. Lewy body, vascular, frontotemporal) based on different data modalities (e.g. grey matter, white matter scans), 2) including clinical care data to capture comorbidities and blood biomarkers that are emerging as a potential scalable tool for dementia prediction, 3) including data from underrepresented groups that may be disproportionately affected by dementia.^30^ Uncovering the key predictors of progression to different dementia subtypes for diverse cohorts is key for tackling the global dementia challenge and developing precision medicine interventions. Our vision is to scale-up our predictive prognostic modelling approach to a responsible AI decision support system that will aid clinicians to assign the right patient at the right time to the right diagnostic and treatment pathway, enhancing clinical management efficiency, patient wellbeing and outcomes.

## Contributors

Conceptualization: LYL, DV, MCB, PT, MM, KZ, JG, CC, BRU, TR, ZK; Data curation: LYL, DV, ZL, ZK, EC; Formal Analysis: LYL, DV, ZL; Funding acquisition: LYL, BRU, ZK; Investigation: MM, EC, CC, TR; Methodology: DV, MCB, PT, JG, ZK; Project administration: LYL, ZK; Resources: PT, MM, GW, EC, CC, BRU, TR, ZK; Software: DV, MCB, PT, KZ, JG; Supervision: LYL, PT, CC, TR, ZK; Validation: LYL, DV, ZL; Visualization: LYL, DV, MCB, ZL, JG; Writing—original draft: LYL, DV, MCB, PT, MM, KZ, GW, EC, CC, BRU, TR, ZK; Writing—review & editing: LYL, DV, MCB, PT, MM, ZL, KZ, JG, GW, EC, CC, BRU, TR, ZK; LYL, DV, MCB, MM, ZL, KZ, JG, EC, CC, BRU, TR and ZK had access and verified the underlying data.

## Data sharing statement

Data and code are available at the University of Cambridge data repository: https://doi.org/10.17863/CAM.108903.

## Declaration of interests

BRU is advisory board member for Eli Lilly, executive member of the Royal College of Psychiatrists., East of England network coordinator for ARUK, East of England clinical lead for dementia for the NIHR clinical research network. All other authors declare no conflicts of interest.
