# Supplementary material for: Robust and interpretable AI-guided marker for early dementia prediction in real-world clinical settings
Source: eClinicalMedicine. 2024 Jul 12;74:102725. doi: 10.1016/j.eclinm.2024.102725 (PMC11701481; doi:10.1016/j.eclinm.2024.102725)
Supplement: Supplementary Information, Figures and Tables [file mmc1.docx]

# **Supplementary Information**

## **PPM training and test samples**

We used data (Figure 1) from: 1) a research cohort (The Alzheimer’s Disease Neuroimaging Initiative, ADNI) for PPM training and within-sample cross-validation (n = 410) and out-of-sample validation (n = 609), 2) two clinical cohorts (Quantitative MRI of Brain Structure and Function in NHS Memory Clinics, QMIN-MC, n = 272; Memory Ageing & Cognition Centre at the National University of Singapore dataset, MACC, n = 605) as independent test datasets for out-of-sample validation.

### *Alzheimer’s Disease Neuroimaging Initiative (ADNI; adni.loni.usc.edu) data*

We trained the PPM on data from individuals diagnosed with mild cognitive impairment (MCI, n = 410) from ADNI2/GO and ADNI3 with longitudinal assessments (i.e. multiple clinical diagnostic assessments within a period of minimum 3 years and a maximum of 4 years; that is, 2-5 assessments). Most participants are White (n = 381), Black (n = 14), Asian (n = 5), and others including Indian/Alaskan, Hawaiian/Other PI and unknown (n = 10) (Figure 1A). MRI data were collected across multiple MRI acquisition sites with 1.5T and 3T scanners (GE, Philips, Siemens scanners). Individuals were characterised as stable MCI (sMCI, n = 290, Age at baseline: mean = 71.45, std = ±7.76 years, Education (number of years): mean = 16.33, std = ±2.78, Sex (M/F) = 118/172) if they consistently received an MCI diagnosis within three years of clinical observation. Individuals who progressed from MCI to AD within 3 years of clinical observation were characterised as progressive MCI (pMCI, n = 120, Age at baseline: mean = 73.66, std = ±6.42, Education (years) mean = 15.94, std = ±2.74 years, Sex (M/F) = 63/57). Individuals in the pMCI group were slightly older than the sMCI group, (t(408) = 2.75, p < 0.05), but the two groups did not differ significantly in years of education, (t(408) = -1.31, p = 0.19). To account for age differences and other potential confounding covariates, we regressed out age, sex, and education from the training features.

To validate the PPM longitudinal predictions on data that were not included in model training, we selected an independent validation sample (n = 609) including individuals with normal cognition (CN, n = 315), stable MCI (sMCI, n = 106) and Alzheimer’s Disease (AD, n = 188). Most participants are White (n = 564), Black (n = 23), Asian (n = 7), and others including Indian/Alaskan, Hawaiian/Other PI and unknown (n = 13). (Figure 1B). These individuals had baseline cognitive assessments and underwent 3T structural MRI scans. The scans were associated with the nearest available clinical assessment (within 6 months of the imaging date). All CN, MCI and some AD (n = 23) individuals had at least one follow-up clinical visit within a 3-year period (1 visit, n = 284; 2 visits, n = 109; 3 visits, n = 6) during which a CDR (Clinical Dementia Rating) test was administered. The rate of CDR decline was calculated as the slope of the line of best fit for the available pairs (t_i_, CDR_i_), where time is measured in years, t = 0 corresponds to the baseline visit, and CDR_i_ was the CDR recorded at time t_i_.

### *Quantitative MRI of Brain Structure and Function in NHS Memory Clinics (QMIN-MC)*

The QMIN-MC sample was recruited from neurology-led and psychiatry-led memory clinics within the NHS (National Health Service in England), covering a broad spectrum of individuals referred to memory services and therefore typical of patient samples in real-life clinical settings (Figure 1C). All patients attending memory clinics who were considered by clinicians to require neuroimaging as part of their diagnostic pathway were considered eligible for the study regardless of diagnosis, age or medical comorbidities. Patients were excluded if they had a contraindication for MRI (e.g. pacemaker or other metal implants).

The QMIN-MC sample comprises data from: a) CUH: Cambridge University Hospital, n = 121, mean age at scanning = 65.11, std = ±9.81 years, Education (years) mean = 13.16, std = ±3.38 years, Sex (M/F) = 59/62; Ethnicity data only available for n = 98: Caucasian (n = 95), Asian/Asian British n = 3; b) CPFT – Cambridgeshire and Peterborough NHS Foundation Trust, n = 106, mean age at scanning = 78.19 std = ±5.99 years, Education (years) mean = 12.08, std = ±3.33 years, Sex (M/F) = 52/54; all participants with ethnicity data available were White (n = 53). Data from a pilot study for QMIN-MC (i.e. Multimodal Imaging in Memory Services, MIMS) were also included (n = 50, mean age = 65.23, std = ±12.47 years, Education (years) mean = 13.10, std = ±3.52 years).

Important information about deprivation data was available for all patients. We utilised postcode information to assign patients to Lower-layer Super Output Areas (LSOAs) and calculate deprivation indices. This was accomplished with an online service (https://imd-by-postcode.opendatacommunities.org/imd/2019) based on the 2019 English indices of deprivation^1^. The resulting dataset included deciles, ranks, and scores for various deprivation measures, including the Index of Multiple Deprivation (IMD) and Income Deprivation Affecting Older People Index. The Index of Multiple Deprivation (IMD) is a composite index combining seven indicators of deprivation, namely income, employment, education, health and disability, crime, barriers to housing and service, and living environment. A higher IMD score suggests a greater degree of disadvantage or deprivation for a particular area. See Noble et al., (2019)^2^ for further information. The Income Deprivation Affecting Older People Index is a specific component of the IMD that captures the level of income deprivation experienced by this vulnerable group. Higher scores indicate higher level of income-related deprivation. The distribution of deprivation indices was examined through the calculation of mean scores, decile rankings, and graphical representations (Figure S1).

### *Memory Ageing & Cognition Centre at the National University of Singapore dataset (MACC)*

Data from 605 individuals with MRI data and cognitive assessment from the Memory Ageing & Cognition Centre at the National University of Singapore (NUS) were used to test the model. Individuals were assigned into four classes based on their clinical diagnosis assessed using a neuropsychological test battery with 6 cognitive domains (Attention, Language, Visuomotor speed, Visuoconstruction, and two Memory domains)^3^ (Figure 1D): Cognitive normal (CN; n = 128, Age: mean = 68.5, std = ±7.5 years, Education years: mean = 9.9, std = ±5.1 years, Sex (M/F) = 58/70); Mild cognitive impairment (MCI mild; n = 160, Age: mean = 71.4, std = ±7.6 years, Education years: mean = 8.1, std = ±4.5 years, Sex (M/F) = 81/79); Moderate cognitive impairment (MCI moderate; n = 116, Age: mean = 75.3, std = ±7.2 years, Education years: mean = 6.7, std = ±5.2 years, Sex (M/F) = 47/69); Alzheimer’s Disease (AD; n = 201, Age: mean = 75.4, std = ±7.1 years, Education years: mean = 4.7, std = ±4.5 years, Sex (M/F) = 63/138). Most patients are Asian (Chinese, n = 505; Malaysian, n = 55; Indian, n = 37; Others, n = 8). Ethics approval for this study was obtained from the National Healthcare Group Domain-Specific Review Board. Written informed consent was obtained in the preferred language of the participants prior to recruitment. The study was conducted in accordance with the Declaration of Helsinki.

**Diagnostic criteria**

*ADNI*: Diagnosis in ADNI was based on MMSE, CDR, and Logical Memory for each diagnostic category as well as Geriatric Depression Scale score less than 6. AD cases were identified based on the following criteria: a) NINCDS/ADRDA criteria for probable AD, b) clinical tests: Clinical Dementia Rating = 0.5 or 1.0, and MMSE score between 20 and 26, c) memory function tests: Abnormal memory function documented by scoring below education adjusted cut-offs on the Logical Memory II subscale (Delayed Paragraph Recall, Paragraph A only) from the Wechsler Memory Scale – Revised (≤ 8 for 16 or more years of education b. ≤ 4 for 8-15 years of education c. ≤ 2 for 0-7 years of education), d) subjective memory concern, e) others: Stability of Permitted Medications for at least 12 weeks. MCI cases were identified based on the following criteria: a) clinical tests: Mini-Mental State Exam score between 24 and 30, b) memory function tests: Abnormal memory function documented by scoring below education adjusted cut-offs on the Logical Memory II subscale (Delayed Paragraph Recall, Paragraph A only) from the Wechsler Memory Scale – Revised (< 11 for 16 or more years of education, ≤ 9 for 8-15 years of education, ≤ 6 for 0-7 years of education), c) ) subjective memory concern. CN cases were identified based on the following criteria: a) clinical tests: Mini-Mental State Exam score between 24 and 30, b) memory function tests: Abnormal memory function documented by scoring below education adjusted cut-offs on the Logical Memory II subscale (Delayed Paragraph Recall, Paragraph A only) from the Wechsler Memory Scale – Revised (9 for 16 or more years of education, >=5 for 8-15 years of education, >=3 for 0-7 years of education), c) With or without subjective memory concern.

#### QMIN-MC: Diagnoses in QMIN-MC were given by neurology or psychiatry consultants experienced in neurodegenerative disease in the context of a memory clinic or memory assessment service, after detailed clinical assessment by a doctor or specialist nurse involving a history, examination and cognitive testing with the Addenbrookes Cognitive Examination (ACE-R, or ACE-III). All diagnoses were discussed at clinical multidisciplinary team meetings. Clinical diagnoses were transcribed to one of 23 diagnostic categories (Table S1), or "other" where an alternate diagnosis was given, "uncertain" where insufficient information to make a diagnosis was available at review but could be available later (e.g. further test results), or "unknown" where a diagnosis could not be reached after investigations were completed.

QMIN-MC patients were split into five groups based on their clinical diagnoses (Table S1): AD (n = 108): patients with a probable diagnosis of Alzheimer’s Disease; MCI (n = 63): patients with Mild Cognitive Impairment; Non-AD Neurodegenerative disorder (n = 43): patients with non-Alzheimer dementia syndromes including vascular dementia, Fronotemporal Dementia, Dementia with Lewy Bodies, Cerebellar syndromes with cognitive impairment, Leucoencephalopathy, Parkinson’s disease dementia, Corticobasal Syndrome, and Unspecified Dementia; Functional/attentional memory symptoms (n = 45): individuals who exhibit impaired memory, but do not show any discernible structural abnormalities in their brain scans, including Functional Memory Disorder, memory symptoms secondary to depression, anxiety or another psychiatric disorder, and memory symptoms from other causes such as chronic pain or medications; Other cognitive disorders (n = 13): patients with non-progressive cognitive disorders such as traumatic brain injury, stroke, and alcohol related cognitive impairment. This study was approved by the East of England - Essex Research Ethics Committee (REC reference: 20/EE/0042, Protocol number: A095373, IRAS project ID: 274332). Cognitive data was collected by NHS memory clinic staff, neuroimaging data was collected at academic or NHS neuroimaging centres. Written consent was obtained prior to recruitment.

*MACC*: Diagnosis in MACC was based on clinical, physical, neuropsychological assessments and neuroimaging at the Yong Loo Lin School of Medicine, National University of Singapore. Relevant demographic and medical information, including vascular risk factors and exclusion factors such as previous head trauma, thyroid disease, non-AD neurodegenerative conditions (e.g., Parkinson’s disease), and psychiatric illnesses, were collected by administering a detailed questionnaire and review of medical records. Further, participants were administered a comprehensive neuropsychological test battery consisting of several domains, namely, executive function, attention, language, visuomotor speed, visuoconstruction, verbal memory and visual memory, along with standard cognitive assessments (Mini-Mental State Examination and Montreal Cognitive Assessment. Diagnoses of cognitive impairment and dementia were made at regular consensus meetings of study clinicians and neuropsychologists. In particular, AD cases were diagnosed using the National Institute of Neurological and Communicative Disorders and Stroke and the Alzheimer’s disease and Related Disorders Association (NINCDS-ADRDA) criteria. Cognitive normal individuals were identified as those with subjective memory complaints, but who were found to be cognitively normal after undergoing objective neuropsychological assessments. Mild cognitive impairment (MCI) individuals did not meet the Diagnostic and Statistical Manual Fourth Edition (DSM-IV) diagnostic criteria for dementia but showed impairment in one or more domains of the neuropsychological battery, as defined by education-adjusted scores ≥ 1.5 standard deviations below normal established means for at least half of the tests for that domain. Further, MCI patients were classified into mild MCI (patients impaired in 1-2 domains of the neuropsychological test battery) and moderate MCI (patients were impaired in 3-6 domains of the neuropsychological test battery, but did not meet criteria for dementia).

## **MRI acquisition**

Structural MRIs for the ADNI samples were acquired at ADNI-GO, ADNI2 and ADNI3 sites equipped with 1.5T and 3T MRI scanners (GE, Philips, Siemens) using a 3D MP-RAGE or IR-SPGR T1-weighted sequences, as described online (http://adni.loni.usc.edu/methods/documents/mri-protocols).

Structural MRIs for the QMIN-MC were collected at a 3T Siemens Magnetom Prisma_fit scanner equipped with a 32-channel head coil at the Wolfson Brain Imaging Centre using MPRAGE sequences (208 slices, TR/TE/TI = 2000/1.95/880 ms, flip angle = 8 degrees, slice thickness = 1mm, voxel size = 1 × 1× 1 mm, FOV = 256 × 256 mm following the UK biobank protocol.

Structural MRIs for the MACC sample were collected on a 3T Siemens Magnetom Tim Trio scanner, equipped with a 32-channel head coil at the Yong Loo Lin School of Medicine, Clinical Imaging Research Centre of National University of Singapore using MPRAGE sequence (192 slices, TR/TE/TI = 2300/1.9/900 ms, flip angle = 9 degrees, slice thickness = 1mm, voxel size = 1 × 1× 1 mm, FOV = 256 × 256 mm^4^.

## **MRI analysis: extracting medial temporal grey matter density**

All imaging pre-processing was performed using Statistical Parametric Mapping 12 in matlab (SPM12, http://www.fil.ion.ucl.ac.uk/spm/) following our previously published pipeline^6^. Structural images were reoriented and segmented into grey matter, white matter and cerebrospinal fluid. We used the DARTEL toolbox^7^ to generate a study-specific template to which all scans were normalised. Individual grey matter segmentation volumes were normalised to MNI space without modulation. The unmodulated values for each voxel represent grey matter density at the voxel location. All images were then smoothed using a 3mm3 isotropic kernel and resliced to MNI resolution 1.5 × 1.5 × 1.5 mm voxel size.

We then generated an index of medial temporal grey matter density (GM density). In particular, subspace learning— that forms the backbone of the GMLVQ methodology— cannot be directly applied in the MRI voxel space due to the prohibitive number of free parameters that would need to be inferred from the sample size used for model training. Therefore, as reported in our previous work^5^, to reduce dimensionality we first performed feature construction in the whole brain T1-weighted MRI voxel space using partial least squares regression with recursive feature elimination (PLSr-RFE) on ADNI (ADNI-GO ADNI-2) data. In particular, we tested for grey matter voxels that predicted memory decline (i.e. annualized change in ADNI memory composite), iteratively removing predictors (voxels) that had weak predictive values and resulting in a bilateral cluster of voxels in MTL that predict cognitive decline. That is, this reduced set of new orthogonal features span the voxel subspace that maximises covariance with the relevant response variable (i.e. memory decline). Using this method, we determined an ROI defined by a matrix of voxel weights in the medial temporal lobe and extracted grey matter density. This grey matter density score was shown to predict memory decline, relate to individual tau burden and discriminate stable vs. progressive MCI^5, 6^. PPM was trained on this reduced set of features (grey matter density score) that is obtained in a data-driven way without the need to infer a prohibitive number of free parameters. We then used this predefined ROI to extract grey matter density from two independent cohorts (i.e. QMIN-MC and MACC) that were used for testing the PPM (i.e. out-of-sample validation).

## **Cognitive data imputation**

Cognitive tests varied across the cohorts used for PPM training and test. In particular, ADNI participants undertook a battery of neuropsychological tests (e.g. Clinical Dementia Rating Scale, CDR^8^, Mini-Mental State Examination, MMSE). QMIN-MC patients were tested with either Addenbrooke’s Cognitive Examination III (ACE-III)^9^ or Addenbrooke’s Cognitive Examination-Revised (ACER)^10^ including five subscales (orientation-attention, memory, verbal fluencies, language, and visuospatial skills). MACC patients were assessed with various cognitive tests including MMSE and Montreal Cognitive Assessment (MoCA). To harmonize cognitive data across cohorts, we implemented a cognitive score imputation method that was previously developed for harmonising data across international ageing and dementia research cohorts^11^. Using this pipeline, we generated harmonized ACER/ACE-III and MMSE scores for the ADNI, QMIN-MC and MACC samples.

In brief, we first pooled cognitive data from international cohorts (ADNI, sample size = 2513, number of neuropsychiatric visits = 10622; MACC, sample size = 636, number of visits = 2553; Neuroimaging of Inflammation in Memory and Related Other Disorders study, NIMROD, sample size = 89, number of visits = 255; Berkeley Aging Cohort Study, BACS, sample size = 188, number of visits = 824; The Australian Imaging, Biomarker & Lifestyle Flagship Study of Ageing, AIBL, sample size = 1820, number of visits = 3920) of varying size and ethnic diversity. Then, we harmonised item-level neuropsychological data using *k*-Nearest Neighbours (*k*-NN), a non-parametric approach to impute missing values by determining the k most similar cases (i.e. neuropsychological visit for any participant) and assigned missing values with the observed value from the closest case (i.e. *k* = 1) or the weighted average of the *k* closest cases. This generated a harmonised battery which covered 125 variables (i.e. item-level neuropsychological assessment), ACE-R scores and MMSE scores for ADNI and MACC. Using the same pipeline, we imputed MMSE scores for patients from QMIN-MC with ACE-III tests using ACE-III total score as ground truth in the K-NN imputation.

## **Predictive Prognostic Model**

### *Learning Vector Quantization*

Learning Vector Quantization (LVQ) is a supervised classification method that iteratively modifies class-specific prototypes to identify boundaries between discrete classes. The LVQ classifiers are defined by a set of vectors known as class prototypes that represent the classes within the input space. During the training phase, the prototypes are updated iteratively based on the training examples. For each training example, the LVQ classifier determines the closest prototype for each class. The prototypes are then adjusted so that the prototype representing the same class as the input example (the closest 'correct' prototype) is moved closer to the example, while the closest prototype among the prototypes representing different classes (the closest 'incorrect' prototype) is moved further away.

During training, for each class, the LVQ algorithm aims to minimize the distances between the training examples of the given class and the prototypes that share the same class label, while maximizing the distances to the prototypes of the other classes. This process helps to form class boundaries with large classification margins.

Once the training is completed, the LVQ classifier can be used for classifying test data. Given a previously unseen input vector, the classifier assigns to it the class label of the closest prototype.

### *The Generalised Matrix LVQ*

Note that in the LVQ family of algorithms the notion of a distance (metric) in the input space place a crucial role. It governs what input points are assigned to which prototypes. The Generalised Metric LVQ (GMLVQ) is an extension of the LVQ algorithm that besides appropriate positioning of the prototypes also learns the metric to be used in the input space that enhances the class separation. The learnt metric is determined through the corresponding metric tensor. The metric tensor is a positive definite matrix Λ feature scaling, as well as axis rotation accounting for the interplay between original input features.

More formally, given a positive definite matrix Λ, Λ > 0, the generalised form of the squared distance between an input vector *x* and a class prototype *w* takes the quadratic form *d_Λ_*(*x*, *w*) = (*x*-*w*)*^T^*\Λ(*x*-*w*).

Positive definiteness of Λ is ensured by defining Λ as Ω^T^Ω, where Ω ∈ *R*^(^*^m^*^×^*^m^*^)^ is a full-rank matrix. It is important to note that only the relative distances of input points to the prototypes are significant. Therefore, the metric tensor can be multiplied by any positive real number without affecting the classifier’s performance. To address this inherent ambiguity and ensure algorithm stability, the metric tensor Λ is normalized after each learning step, such as by maintaining the trace fixed throughout the learning process.

It is important to note that for the classification purposes only relative distances between input points and prototypes are relevant. Indeed, the metric tensor can be scaled by any positive real number without affecting the classifier's performance. To address the inherently ill-posed nature of the model fitting and ensure algorithm stability, Λ should undergo normalization after each learning step ensuring unit trace, ∑*_i_*Λ*_i_*_,_*_i_* =1.

Using the steepest descent method, the cost function to be minimised through online learning is

$$f_{GMLVQ}=\sum_{i=1}^{n} \left( \varphi{(\mu}_{\Lambda}{(x}_{i}) \right)$$

where

$$\mu_{\Lambda}{(x}_{i})= \frac{d\Lambda\left( xi, w+ \right)-d\Lambda(xi, w-)}{d\Lambda\left( xi, w+ \right)+d\Lambda(xi, w-)}$$

where *φ* is a monotonic identity function (in our case identify *φ*(*l*) = *l*), *d*_Λ_(*x_i_*, *w*^+^) and *d*_Λ_(*x_i_*, *w*^-^) are the distances between the sample vector *x_i_* and the closest correct and incorrect prototypes, respectively. We assessed the model performance by classification accuracy, true positive rate, true negative rate and macro averaged error (MAE).

A challenge in averaging symmetric matrices is maintaining positive definiteness. Metric tensors are expected to be positive definite, meaning all their eigenvalues are positive. It is important to ensure the averaged matrix remains positive definite.

Further, we introduced ensemble learning, combining multiple models to make more accurate predictions, and enhance robustness. In particular, for each cross-validation, we split the data into training-fold and test-fold. To mitigate any potential biases due to class imbalance in the dataset (sMCI (n = 290), pMCI (n = 120)), we resampled the data to generate balanced classes. For each training-fold, we repeatedly (n = 400) randomly down-sampled the majority class (i.e. sMCI) to match the size of the minority class (i.e. pMCI). For each resampling, we generated a new GMLVQ model that was trained on the resampled data in the training-fold (i.e. the model learned the metric tensor and prototype locations specific to each resampled training set) and validated on the test-fold. From this ensemble of models, we selected the top 20% (n = 80) classifiers based on their training set performance. Note that this selection could not be done based on the out-of-sample performance, as this could lead to biased models; that is, the ensemble members would be selected on the same sets the ensemble generalization performance is assessed on. However, as we employ one prototype per class, under the global metric tensor, all classification decision boundaries are linear. Hence, the risk of overfitting the training set is minimized and the training set classification accuracies are reasonable proxies for the out-of-sample ones: the ensemble member selection can be performed on the training sets without needing to compromise the out-of-sample sets. We then estimated the class balanced accuracy based on a) majority vote, i.e. the class label that receives the most votes from the ensemble models is selected as the final prediction^12^, b) the average performance across the selected classifiers^13^. This ensemble learning approach with cross-validation helps mitigate for potential individual model biases, resulting in more robust and accurate predictions.

### *GMLVQ-Scalar Projection*

Moving beyond binary classifications, we extended the GMLVQ framework to generate continuous predictions from baseline cognitive data and structural MRI data (temporal lobe GM density). In particular, we employed GMLVQ-Scalar Projection^5^ that extracts distance information from the sample vector and the learnt prototypes (representing stable and progressive MCI). GMLVQ-Scalar Projection measures the distance in the learnt space, after applying the learnt metric tensor, between an individual and the prototype representing stable MCI along the direction separating stable and progressive MCI (the line connecting stable and progressive class prototypes). We extracted the scalar projection using the average prototypes and metric tensors of the selected top 20% classifiers to capture robust information across the ensemble of trained classifiers (note, majority voting does not support extraction of scalar projection).

In particular, following the learning process in GMLVQ, we transformed the sample vector *x* and prototypes *w*_(_*_stable_*_,_ *_progressive_*_)_ into the learned space using the metric tensor Λ. The geometric effect of the metric tensor on the original data vectors *x* can be interpreted as change of basis and rescaling: *X_i_* = Λ^1/2^*x_i_*. Under such transformation, the learnt distances between data points *x_i_* are equal to the usual Euclidean distance between the transformed points *x_i_*. Hence, we accordingly transformed the data points and class prototypes: $W_{(stable, progressive)}$ = Λ^1/2^ $w_{(stable,progressive)}$.

To further analyze the separation of each vector *x* from the prototype *W* along the given direction, we centered the coordinate system on the prototype p and calculated the orthogonal projection of each vector x onto the direction vector. The direction vector is defined as the difference between the stable and the progressive prototypes:

$$Projection= \frac{\vec{X_{l}W_{stable}} \cdot\vec{W_{progressive}W_{stable}}}{\left| \vec{W_{progressive}W_{stable}} \right|}$$

To normalize the projections with respect to the position of the prototype $W_{progressive}$, we divided each projection by the norm of the direction vector. This normalization step allows us to determine the relative separation of a test point from the stable prototype.

$$Scalar Projection= \frac{\vec{X_{l}W_{stable}} \cdot\vec{W_{progressive}W_{stable}}}{\left| \vec{W_{progressive}W_{stable}} \right|^{2}}$$

The resulting value indicates the separation of a test point from the prototype $W_{stable}$ along the direction of $\vec{W_{progressive}W_{stable}}$. A large positive value suggests a significant separation from the stable prototype $W_{stable}$in the direction, while a large negative value indicates a substantial separation in the opposite direction. A value of 1 signifies that a sample is incident to the prototype $W_{progressive}$, while a value of 0 indicates that a sample is incident to the prototype $W_{stable}$, representing the stable class. The decision boundary separating the two classes within the binary classification framework is located at a value of 0.5. The scalar projection, obtained by performing these calculations, yields a large positive value for progressive MCI (pMCI) individuals and a zero or negative value for stable MCI (sMCI) individuals. This scalar projection index serves as a discriminative indicator of the classification task, where higher positive values correspond to a higher likelihood of being classified as pMCI. That is, the scalar projection index captures information about how far an individual is from the sMCI prototype, serving as an individualised PPM-derived prognostic index. We have previously shown that this index relates significantly to the rate of memory decline, allowing us to estimate how fast an individual progresses from MCI to AD^6^.

We next used multinomial logistic regression to test the relationship of scalar projection index to the rate of cognitive decline (i.e. future MMSE slope) and determine quartile classes (based on boundaries) that represent different levels of progression. We estimated the probabilities of each quartile class for a range of boundary values and identified the boundaries based on the quartile class with the highest probability at each value. The lower boundary (at the 20^th^ percentile of the future MMSE slope) indicates individuals who are more likely to experience slower progression (slowly progressive). Conversely, the higher boundary (at the 60^th^ percentile of the future MMSE slope) indicates individuals who are more likely to experience faster progression (rapidly progressive). This multinomial logistic regression model allows us to stratify individuals based on their PPM-derived prognostic index (i.e. the scalar projection score) and future MMSE slope.

## **Statistical analyses**

We tested for data normality using the Shapiro-Wilk test. As the data were not normally distributed (ADNI, PPM trained on MRI and cognitive data, W(609) = 0.881, p < 0.001; ADNI, PPM trained on cognitive data, W(609) = 0.862, p < 0.001; QMIN-MC, PPM trained on MRI and cognitive data, W(272) = 0.980, p < 0.001; QMIN-MC, PPM trained on cognitive data , W(272) = 0.967, p < 0.001; MACC, PPM trained on MRI and cognitive data, W(605) = 0.959, p < 0.001; MACC, PPM trained on cognitive data, W(605) = 0.954, p < 0.001), we used Kruskal-Wallis H test Bonferroni corrected (p < 0.05) to examine the differences in the PPM-derived prognostic index across the different patient groups. We applied Bonferroni correction (p < 0.05) to correct for multiple comparisons. The analysis was conducted using SPSS version 28.0 (IBM Corp., Armonk, NY, USA). We used the DeLong test to compare AUCs (area under the ROC curves) across models.

We used Spearman’s rank correlation to test whether the relationship between the PPM-derived prognostic index and the rate of future cognitive decline (CDR change) was significant, using MATLAB’s *corrcoef* function. To test whether the relationship between the PPM-derived prognostic index and rate of future cognitive decline significantly differed between models i.e. PPM trained on MRI and cognitive data vs. PPM trained on cognitive data alone, we used Steiger Z^14,15^.

To investigate whether the PPM-derived prognostic score based on baseline data predicts future cognitive decline, we conducted a survival analysis^16^ using Python lifelines package^17^ on the MACC cohort, as most patients have longitudinal diagnoses (1 visit, n = 38; 2 visits, n = 49; 3 visits, n = 48; 4 visits, n = 101; 5 visits, n = 112; 6 visits, n = 257). We excluded patients with only one visit and those with AD diagnosis at first assessment, resulting in total of 387 patients. As the outcome variable for this analysis is an event^16^ (i.e. conversion to AD), each patient was labelled per visit either as ‘converted to AD’ (i.e. the patient received diagnosis of AD, n = 72) or ‘non-converted to AD’ (i.e. the patient remained healthy or MCI, n = 315) based on their clinical diagnosis at the time of a given visit. We then stratified MACC patients into three groups based on: a) PPM-derived prognostic index at baseline (i.e. first visit with imaging data): stable, n = 189; slowly progressive, n = 111; rapidly progressive, n = 87), b) clinical diagnosis at baseline (mild MCI, n = 153; moderate MCI, n = 108). For each group (stable, slowly progressive, rapidly progressive), we estimated the probability of non-conversion to AD as a function of time using clinical observations and the Kaplan-Meier estimator^18^. We used the logrank test^19^ (https://lifelines.readthedocs.io/en/latest/lifelines.statistics.html) to compare the relative risk of ‘conversion to AD’ for patient groups given different prognostic labels by the model. We used a multivariate Cox proportional hazards regression analysis^20^ to identify independent predictors of ‘conversion to AD’ with hazard ratios at 95% confidence intervals (CIs).

# **Supplementary Figures**


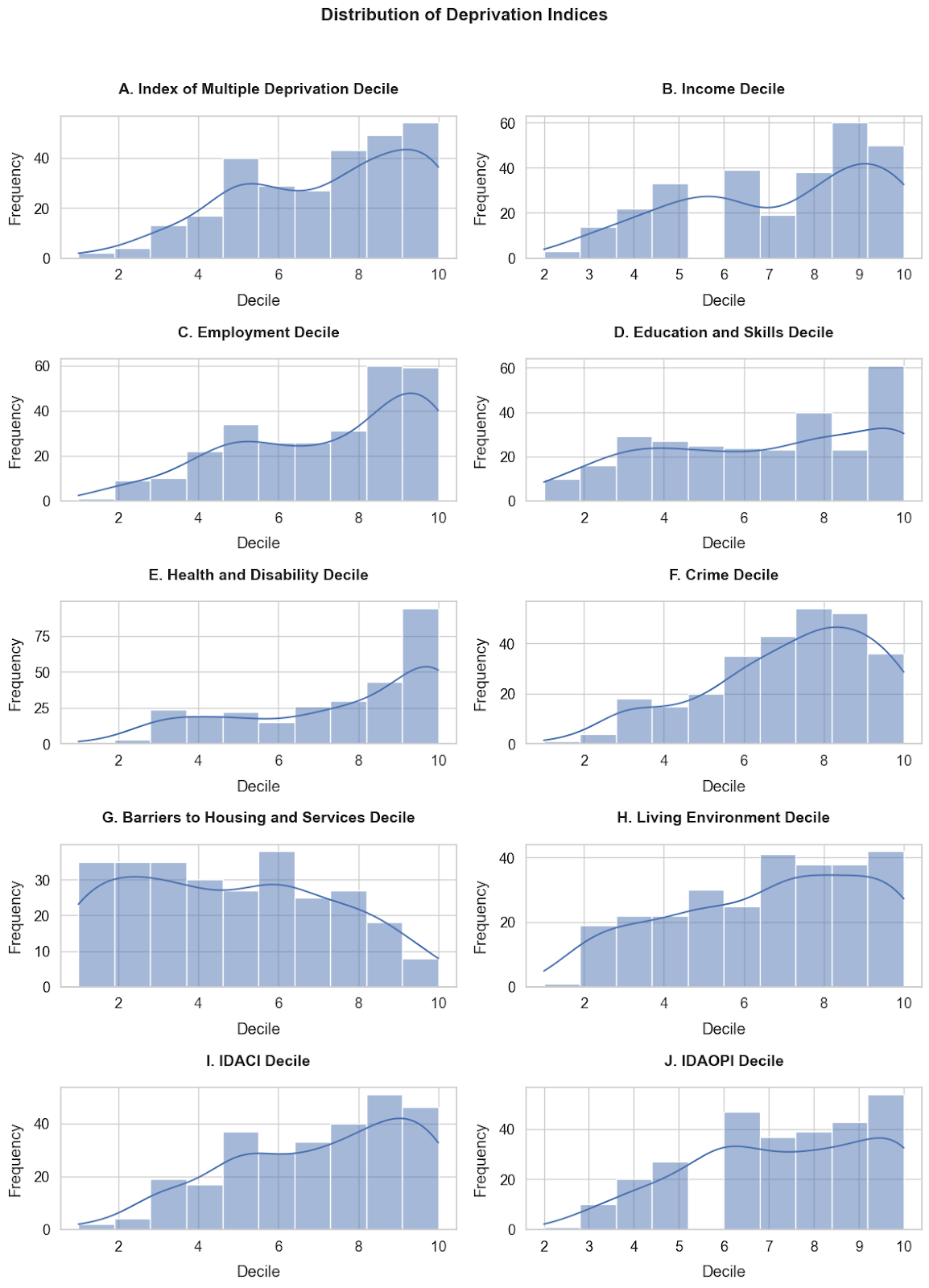


**Figure S1. Distribution of Deprivation Indices for QMIN-MC patients.** Histograms represent the distribution of deprivation indices, where lower deciles indicate higher levels of deprivation. Each plot (labelled A to J) corresponds to a specific deprivation index, indicating the distribution and variation of deprivation indices across the dataset. IDACI = The Income Deprivation Affecting Younger People Index; IDAOPI = The Income Deprivation Affecting Older People Index


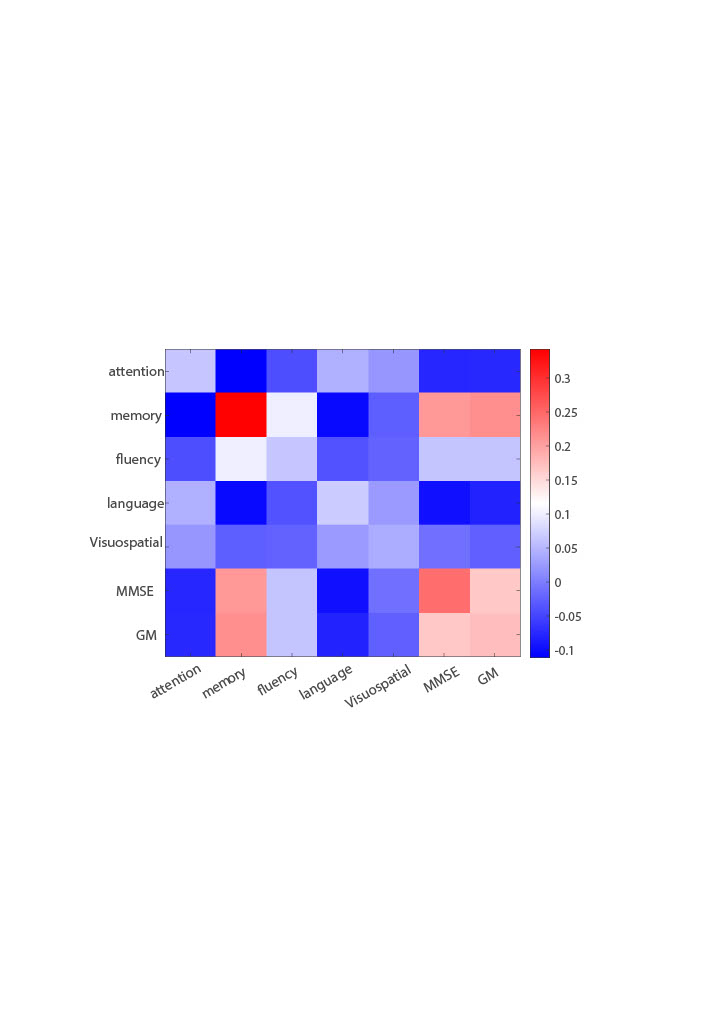


**Figure S2. PPM Metric Tensor for sMCI vs. pMCI classification**: PPM metric tensor generated using different ACER tasks/items (attention, memory, fluency, language, visual), MMSE, and medial temporal grey matter density. The colour scale represents predictive values for each cell in the metric tensor, with diagonal terms summing 1. The diagonal terms show stronger contribution of ACER memory (0.27 [0.26, 0.28]), MMSE (0.17 [0.16, 0.18]), and grey matter density (0.17 [0.16, 0.18]) compared to ACE-R Language (0.12 [0.11, 0.13]), ACE-R Attention (0.11 [0.11, 0.12]), ACE-R Visuospatial (0.08 [0.08, 0.08]), ACE-R Fluency (0.08 [0.08, 0.08]). Positive off-diagonal terms indicate interactions between these features.


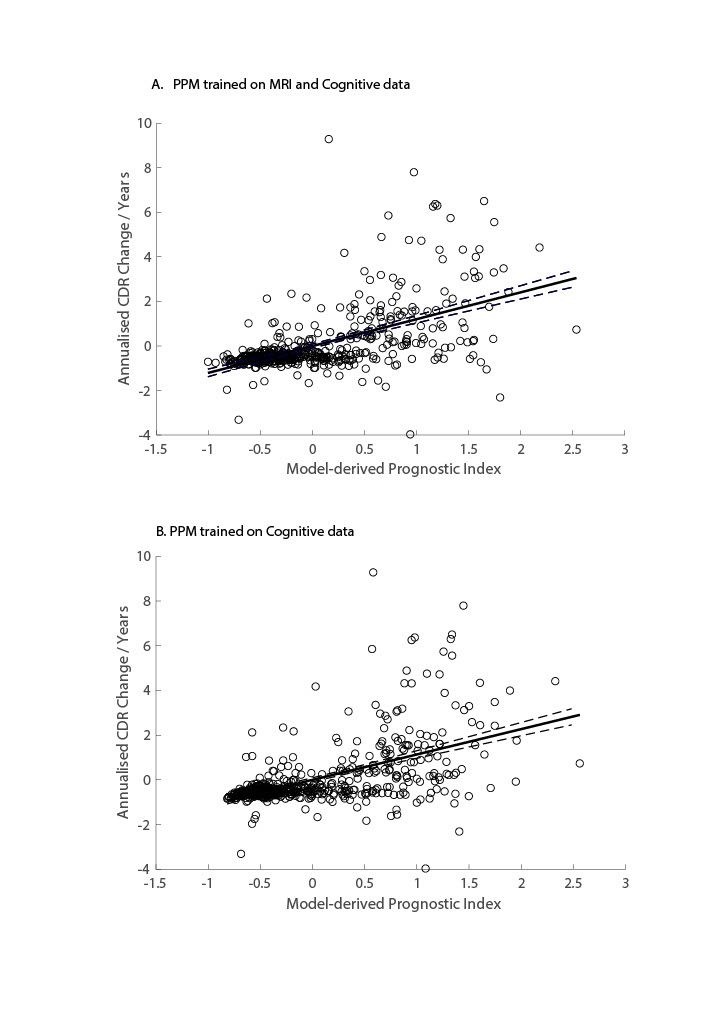


**Figure S3. PPM-derived index correlation against CDR change.** PPM-derived prognostic index corelates significantly with annualized CDR change when **A**. PPM was trained with MRI and cognitive data, **B**. PPM was trained with cognitive data alone. The dashed lines represent the 95% confidence intervals for the regression line in bold.

# **Supplementary Tables**

**Table S1.** QMIN-MC diagnostic labels and correspondence to patient groups included in the PPM.

| **QMIN-MC diagnosis** | **QMIN-MC patient groups** |
| --- | --- |
| Alzheimer's disease | AD |
| Alzheimer's disease - Posterior Cortical Atrophy | AD |
| Alzheimer's disease - Logopenic aphasia | AD |
| Mild Cognitive Impairment | MCI |
| Vascular dementia | Non-AD neurodegenerative disorders |
| Mixed dementia (Alzheimer's disease and vascular dementia) | Non-AD neurodegenerative disorders |
| Alcohol related dementia | Non-AD neurodegenerative disorders |
| Korsakoff's syndrome | Other |
| Depression | Other |
| Anxiety | Other |
| Functional/Attentional Memory Disorder | Functional/Attentional Memory Symptoms |
| Dementia with Lewy Bodies | Non-AD neurodegenerative disorders |
| Parkinson's disease | Non-AD neurodegenerative disorders |
| Parkinson's disease dementia | Non-AD neurodegenerative disorders |
| Frontotemporal dementia - behavioural variant | Non-AD neurodegenerative disorders |
| Frontotemporal dementia - non-fluent variant aphasia | Non-AD neurodegenerative disorders |
| Frontotemporal dementia - semantic dementia | Non-AD neurodegenerative disorders |
| Progressive Supranuclear Palsy | Non-AD neurodegenerative disorders |
| Corticobasal Syndrome | Non-AD neurodegenerative disorders |
| Multiple System Atrophy | Non-AD neurodegenerative disorders |
| Motor Neurone Disease | Non-AD neurodegenerative disorders |
| Traumatic brain injury | Other |
| Stroke | Other |

**Table S2.** PPM class-balanced accuracy (mean and std in brackets), sensitivity, specificity, AUC , F1-score , precision, Recall, Precision-Recall AUC for sMCI vs. pMCI classification for ADNI data (n = 410; pMCI = 120; sMCI = 290) when different input features are used for PPM training and test. Numbers in brackets indicate 95% confidence intervals.

| **Feature set** | **Performance Majority voting** | **Performance Average of classifiers** |
| --- | --- | --- |
| ACER Mem, MMSE, GM density | Accuracy: 81.66% [81.23,82.09]  Sensitivity: 82.38% [81.69, 83.07]  Specificity: 80.94% [80.58, 81.29]  AUC: 0.84 [0.83, 0.84]  F1-score: 0.82 [0.81, 0.82]  Precision: 80.94% [80.58, 81.29]  Recall: 82.38% [81.69, 83.07]  Precision-Recall AUC: 0.83 [0.82, 0.83] | Accuracy: 81.40 % [80.22,82.58]  Sensitivity: 81.99% [79.91,84.06]  Specificity: 80.81% [79.45,82.18]  AUC: 0.84 [0.83,0.85]  F1-score: 0.80 [0.78,0.82]  Precision: 79.26% [77.08,81.43]  Recall:81.99% [79.91,84.06]  Precision-Recall AUC: 0.82 [0.81,0.83] |
| ACER Mem, MMSE | Accuracy: 80.03% [79.71, 80.35]  Sensitivity: 80.58% [79.79, 81.38]  Specificity: 79.48% [78.86, 80.10]  AUC: 0.83 [0.82, 0.83]  F1-score: 0.80 [0.79, 0.80]  Precision: 79.48% [78.86,80.10]  Recall: 80.58% [79.79, 81.38]  Precision-Recall AUC: 0.82 [0.81,0.82] | Accuracy: 80.00% [78.46,81.18]  Sensitivity: 81.05% [77.94, 83.14]  Specificity: 79.10% [78.10, 80.10]  AUC: 0.83 [0.82,0.84]  F1-score: 0.77 [0.75, 0.80]  Precision: 75.66% [72.93 78.39]  Recall: 81.05% [77.94, 83.14]  Precision-Recall AUC: 0.82 [0.81,0.83] |
| ACER Mem | Accuracy: 78.68% [78.53, 78.82]  Sensitivity: 79.83% [79.58, 80.08]  Specificity: 77.52% [77.26, 77.77]  AUC: 0.81 [0.80,0.81]  F1-score: 0.76 [0.79, 0.79]  Precision: 77.52% [77.26, 77.77]  Recall: 79.96% [0.79, 0.79]  Precision-Recall AUC: 0.81 [0.80,0.81] | Accuracy: 78.75% [77.38,80.13]  Sensitivity: 79.96% [77.55,82.37]  Specificity: 77.54% [76.23,78.86]  AUC: 0.81 [0.80,0.82]  F1-score: 0.76 [0.74, 0.78]  Precision: 74.36% [71.90, 76.82]  Recall: 79.96% [77.55,82.37]  Precision-Recall AUC: 0.80 [0.79,0.81] |
| GM density | Accuracy: 73.77% [73.44, 74.11]  Sensitivity: 70.17% [69.79, 70.54]  Specificity: 77.38% [76.83, 77.93]  AUC: 0.78 [0.77,0.78]  F1-score: 0.74 [0.73, 0.74]  Precision: 77.38% [76.83, 77.93]  Recall: 70.17% [69.79, 70.54]  Precision-Recall AUC: 0.79 [0.78,0.80] | Accuracy: 73.61%; [72.09,75.13]  Sensitivity: 69.76%; [66.97, 72.55]  Specificity: 77.39%; [76.05, 78.87]  AUC: 0.78 [0.77,0.79]  F1-score: 0.69 [0.67,0.72]  Precision: 69.32% [66.59,72.06]  Recall: 69.76% [66.97,72.55]  Precision-Recall AUC: 0.72 [0.71,0.73] |
| MMSE | Accuracy: 74.44% [74.30, 74.58]  Sensitivity: 74.33% [74.08, 74.58]  Specificity: 74.55% [74.25, 74.85]  AUC: 0.76 [0.75,0.76]  F1-score: 0.74 [0.74, 0.75]  Precision: 74.55% [74.25, 74.85]  Recall: 74.33% [74.08, 74.58]  Precision-Recall AUC: 0.76 [0.75,0.76] | Accuracy: 74.51% [73.22,75.80] Sensitivity: 74.46% [72.16,76.77]  Specificity: 74.55% [73.09,76.03]  AUC: 0.76 [0.75,0.77]  F1-score: 0.73 [0.71, 0.75]  Precision: 72.19% [70.01,74.34]  Recall: 74.46% [72.16,76.77]  Precision-Recall AUC: 0.75 [0.74,0.76] |
| ACER attention, ACER fluency, ACER language, ACER visual | Accuracy: 75.78% [75.45, 76.11]  Sensitivity: 70.83% [69.99, 71.68]  Specificity: 80.72% [80.27, 81.18]  AUC: 0.81 [0.80,0.81]  F1-score: 0.75 [0.75, 0.76]  Precision: 80.72% [80.27, 81.18]  Recall: 70.01% [67.50,72.52]  Precision-Recall AUC: 0.81 [0.80,0.81] | Accuracy: 75.07% [73.75,76.38]  Sensitivity: 70.01% [67.50,72.52]  Specificity: 80.72% [78.85,81.40]  AUC: 0.81 [0.80,0.82]  F1-score: 0.70 [0.68, 0.73]  Precision: 77.77% [67.50, 72.52]  Recall: 70.01% [67.50,72.52]  Precision-Recall AUC: 0.79 [0.78,0.80] |

# **Reference**

1. Noble, S. *et al.* The English indices of deprivation 2019. *CLG Ministry of Housing, Editor. London* (2019).

2. Noble, M., Wright, G., Smith, G. & Dibben, C. Measuring Multiple Deprivation at the Small-Area Level. *Environment and Planning A: Economy and Space* **38**, 169–185 (2006).

3. Narasimhalu, K. *et al.* Severity of CIND and MCI predict incidence of dementia in an ischemic stroke cohort. *Neurology* **73**, 1866–1872 (2009).

4. Zhang, L. *et al.* Cerebral microinfarcts affect brain structural network topology in cognitively impaired patients. *Journal of Cerebral Blood Flow & Metabolism* **41**, 105–115 (2021).

5. Giorgio, J. *et al.* A robust and interpretable machine learning approach using multimodal biological data to predict future pathological tau accumulation. *Nat Commun* **13**, 1887 (2022).

6. Giorgio, J., Landau, S. M., Jagust, W. J., Tino, P. & Kourtzi, Z. Modelling prognostic trajectories of cognitive decline due to Alzheimer’s disease. *Neuroimage Clin* **26**, (2020).

7. Ashburner, J. A fast diffeomorphic image registration algorithm. *Neuroimage* **38**, 95–113 (2007).

8. Morris, J. C. Clinical Dementia Rating: A Reliable and Valid Diagnostic and Staging Measure for Dementia of the Alzheimer Type. *Int Psychogeriatr* **9**, 173–176 (1997).

9. Calderón, C., Beyle, C., Véliz-García, O. & Bekios-Calfa, J. Psychometric properties of Addenbrooke’s Cognitive Examination III (ACE-III): An item response theory approach. *PLoS One* **16**, e0251137 (2021).

10. Mioshi, E., Dawson, K., Mitchell, J., Arnold, R. & Hodges, J. R. The Addenbrooke’s Cognitive Examination Revised (ACE‐R): a brief cognitive test battery for dementia screening. *Int J Geriatr Psychiatry* **21**, 1078–1085 (2006).

11. Giorgio, J. *et al.* A robust harmonization approach for cognitive data from multiple aging and dementia cohorts. (2023).

12. Lam, L. & Suen, S. Y. Application of majority voting to pattern recognition: an analysis of its behavior and performance. *IEEE Transactions on Systems, Man, and Cybernetics - Part A: Systems and Humans* **27**, 553–568 (1997).

13. Kuncheva, L. I. *Combining Pattern Classifiers: Methods and Algorithms*. (John Wiley & Sons, 2014).

14. Steiger, J. H. Tests for comparing elements of a correlation matrix. *Psychol Bull* **87**, 245–251 (1980).

15. Lenhard, W. & Lenhard, A. Hypothesis Tests for Comparing Correlations. https://www.psychometrica.de/correlation.html doi:10.13140/RG.2.1.2954.1367.

16. Collett, D. *Modelling Survival Data in Medical Research*. (Chapman and Hall/CRC, Boca Raton, 2023). doi:10.1201/9781003282525.

17. Davidson-Pilon, C. lifelines: survival analysis in Python. *J Open Source Softw* **4**, 1317 (2019).

18. Kaplan, E. L. & Meier, P. Nonparametric Estimation from Incomplete Observations. *J Am Stat Assoc* **53**, 457–481 (1958).

19. Peto, R. *et al.* Design and analysis of randomized clinical trials requiring prolonged observation of each patient. I. Introduction and design. *Br J Cancer* **34**, 585–612 (1976).

20. Cox, D. R. Regression Models and Life‐Tables. *Journal of the Royal Statistical Society: Series B (Methodological)* **34**, 187–202 (1972).
